# Mutagenicity of acrylamide and glycidamide in human *TP53* knock-in (Hupki) mouse embryo fibroblasts

**DOI:** 10.1007/s00204-020-02878-0

**Published:** 2020-09-04

**Authors:** Lisa Hölzl-Armstrong, Jill E. Kucab, Sarah Moody, Edwin P. Zwart, Lucie Loutkotová, Veronica Duffy, Mirjam Luijten, Gonçalo Gamboa da Costa, Michael R. Stratton, David H. Phillips, Volker M. Arlt

**Affiliations:** 1 Department of Analytical, Environmental and Forensic Sciences, MRC-PHE Centre for Environment and Health, King’s College London, London, SE1 9NH UK; 2 Cancer, Ageing and Somatic Mutation, Wellcome Trust Sanger Institute, Hinxton, CB10 1SA UK; 3 Center for Health Protection, National Institute for Public Health and the Environment (RIVM), Bilthoven, 3720 The Netherlands; 4 Division of Biochemical Toxicology, National Center for Toxicological Research, U.S. Food and Drug Administration, Jefferson, AR 72079 USA; 5 Present Address: Covance Inc., Salt Lake City, Utah 84124 USA; 6 Present Address: Toxicology Department, GAB Consulting GmbH, 69126 Heidelberg, Germany

**Keywords:** *TP53*, Mutation, Whole-genome sequencing, Dietary carcinogen, Acrylamide, DNA adducts

## Abstract

Acrylamide is a suspected human carcinogen formed during high-temperature cooking of starch-rich foods. It is metabolised by cytochrome P450 2E1 to its reactive metabolite glycidamide, which forms pre-mutagenic DNA adducts. Using the human *TP53* knock-in (Hupki) mouse embryo fibroblasts (HUFs) immortalisation assay (HIMA), acrylamide- and glycidamide-induced mutagenesis was studied in the tumour suppressor gene *TP53*. Selected immortalised HUF clones were also subjected to next-generation sequencing to determine mutations across the whole genome. The *TP53*-mutant frequency after glycidamide exposure (1.1 mM for 24 h, *n* = 198) was 9% compared with 0% in cultures treated with acrylamide [1.5 (*n* = 24) or 3 mM (*n* = 6) for 48 h] and untreated vehicle (water) controls (*n* = 36). Most glycidamide-induced mutations occurred at adenines with A > T/T > A and A > G/T > C mutations being the most common types. Mutations induced by glycidamide occurred at specific *TP53* codons that have also been found to be mutated in human tumours (i.e., breast, ovary, colorectal, and lung) previously associated with acrylamide exposure. The spectrum of *TP53* mutations was further reflected by the mutations detected by whole-genome sequencing (WGS) and a distinct WGS mutational signature was found in HUF clones treated with glycidamide that was again characterised by A > G/T > C and A > T/T > A mutations. The WGS mutational signature showed similarities with COSMIC mutational signatures SBS3 and 25 previously found in human tumours (e.g., breast and ovary), while the adenine component was similar to COSMIC SBS4 found mostly in smokers’ lung cancer. In contrast, in acrylamide-treated HUF clones, only culture-related background WGS mutational signatures were observed. In summary, the results of the present study suggest that glycidamide may be involved in the development of breast, ovarian, and lung cancer.

**Electronic supplementary material:**

The online version of this article (10.1007/s00204-020-02878-0) contains supplementary material, which is available to authorized users.

## Introduction

The suspected dietary carcinogen acrylamide is formed in starch-rich food heated above 120 °C (e.g., potato crisps) and it is also present in tobacco smoke (EFSA 2015). In the human body, acrylamide is metabolised by cytochrome P450 (CYP) 2E1 to glycidamide, which can form adducts with DNA. The main DNA adduct is N7-(2-carbamoyl-2-hydroxyethyl)guanine (N7-GA-Gua), which is formed along with two minor adducts N3-(2-carbamoyl-2-hydroxyethyl)adenine (N3-GA-Ade) and N1-(2-carboxy-2-hydroxyethyl)-2′-deoxyadenosine (N1-GA-dA) that lead to base substitutions such as G > T/C > A, A > T/T > A, and A > G/T > C (Supporting Fig. S1) (Besaratinia and Pfeifer 2007; Gamboa da Costa et al. 2003). Acrylamide induces tumours at various organ sites in rodents (e.g., mammary glands, lung) after biotransformation to glycidamide (Beland et al. 2015; EFSA 2015). Epidemiological studies have been inconsistent concerning acrylamide’s role in human cancer development. The International Agency for Research on Cancer (IARC) has classified it as Group 2A probable human carcinogen (IARC 1994), while glycidamide has not yet been assessed. However, both compounds have been listed as high priority agents for the 2020–24 assessment by an IARC Monograph Working Group, highlighting the importance of re-evaluating the association of acrylamide with human carcinogenesis (IARC Monographs Priorities Group 2019).

Various environmental carcinogens leave fingerprints in our genome, notably mutations in the *TP53* gene (Hainaut and Pfeifer 2016). For instance, *TP53* carries G > T/C > A transversions in smokers’ lung cancer associated with exposure to polycyclic aromatic hydrocarbons such as benzo[*a*]pyrene (BaP) or A > T/T > A transversions in urothelial carcinomas associated with aristolochic acid I (AAI) exposure (Kucab et al. 2010). A useful tool to study *TP53* mutations is the human *TP53* knock-in (Hupki) mouse, which has human exons 4–9 of the *TP53* gene in place of the murine exons. The Hupki mouse embryo fibroblasts (HUFs) immortalisation assay (HIMA) can be used to create and select *TP53* mutants following mutagen treatment (Hölzl-Armstrong et al. 2019). DNA of those immortalised HUF mutants can be subjected to Sanger sequencing to acquire information about *TP53* mutations induced by mutagens. As *TP53* is the most commonly mutated gene in human cancer (Kucab et al. 2010), a database that lists almost 30,000 mutations in *TP53* from human tumours has been curated by IARC (www.p53.iarc.fr). Data gained in the HIMA can be compared with information found in this database. Indeed, the *TP53* mutation patterns of several environmental carcinogens (e.g., BaP and AAI) were replicated using the HIMA (Hölzl-Armstrong et al. 2019).

Until recently, the analysis of carcinogen-exposed clones immortalised in the HIMA was restricted to a single gene (i.e., *TP53*) limiting the scope and statistical power of these experiments. However, with the recent advent of next-generation sequencing (NGS), it is now possible to gain information about exome-wide mutations induced by carcinogens or even across the whole genome (Hölzl-Armstrong et al. 2019). Using non-negative matrix factorisation to dissect the complex dataset gained from whole-genome sequencing (WGS) of human tumours, mutational signatures defined by single base substitutions (SBS) and their contribution to the respective tumour type can be acquired (Alexandrov et al. 2013). Some recent studies have also applied this approach to carcinogen-exposed experimental systems (e.g., cultured mammalian cells) (Huang et al. 2017; Kucab et al. 2019; Nik-Zainal et al. 2015). The Catalogue Of Somatic Mutations In Cancer (COSMIC) Database (https://cancer.sanger.ac.uk/cosmic/signatures) currently lists 49 SBS mutational signatures from the whole genomes or exomes of ~ 24,000 tumours (Alexandrov et al. 2020). Subjecting carcinogen-exposed immortalised HUFs to whole-exome sequencing (WES) (Olivier et al. 2014) and WGS (Nik-Zainal et al. 2015) extracted mutational signatures that showed high similarity to some mutational signatures observed in the human tumours where exposure to the respective carcinogen (e.g., BaP and AAI) has been demonstrated.

In the present study, primary HUFs were treated with acrylamide and glycidamide to perform the HIMA to understand if they induce characteristic *TP53* mutations that can help to study cancer aetiology by using the IARC TP53 mutation database. Furthermore, DNA from immortalised HUFs was subjected to NGS to investigate whether WGS mutational signatures specific to acrylamide or glycidamide exposure are related to COSMIC signatures in human tumours. Assay treatment conditions were optimised prior to starting the HIMA by assessing cytotoxicity and induction of DNA damage response (DDR) markers in primary HUFs after exposure to acrylamide and glycidamide. N7-GA-Gua adduct formation was evaluated to determine if pre-mutagenic damage occurred and if subsequently *TP53* and whole-genome mutation types could be linked to DNA damage. The *lacZ* mutation assay was performed to estimate the *TP53-mutant* frequency prior to the HIMA as it has been shown recently that mutagenicity in the *lacZ* reporter gene can be used as a predictor of *TP53* mutagenicity in HUFS (Kucab et al. 2016).

## Materials and methods

### Carcinogens

Acrylamide (≥ 98%, CAS number 79-06-1; #01700) and glycidamide (≥ 95 %; CAS number: 5694-00-8; #4704) were purchased from Sigma-Aldrich (St. Louis, Missouri, USA) and dissolved in nuclease-free water to a stock concentration of 2 M. Sterile-filtrated aliquots were stored at – 20 °C. 3-Nitrobenzanthrone (3-NBA) was synthesised as described previously (Arlt et al. 2002), and stored in aliquots at – 20 °C as a 2 mM stock solution in dimethyl sulfoxide (DMSO).

### Isolation of primary mouse embryo fibroblasts

Primary HUFs were isolated from 13.5-day-old embryos of *Hupki*^+*/*+^*; lacZ*^+^*; Xpa*^+*/*+^ (Arg/Arg codon 72) mice according to a published procedure (Kucab et al. 2015). In addition to the *Hupki* allele, a knock-in allele harbouring exons 4–9 of the human *TP53* gene, the mice harbour the pUR288 plasmid containing a bacterial *lacZ* mutation marker gene, which is integrated in ~ 20 tandem copies per haploid genome into the chromosome. The mice were bred by crossing homozygous Hupki mice of the 129/Sv background with transgenic *Xpa*^±^ mice of the C57B1/6 background containing the pUR288 plasmid. Genotyping was performed where needed as described previously (Kucab et al. 2015). Stocks were prepared on day 3 of HUFs being in culture and were designated as passage 0. More information about the Hupki/XPA/lacZ [B6; 129-Trp53tm1holl-Xpatm1Hvs-Tg(pUR288)1Vij] strain can be found at the European MouseMutant Archive (EMMA: www.infrofrontier.eu) using the EMMA ID EM:08137.

### Culture of HUFs

Cells were cultured in growth medium (Dulbecco’s Modified Eagle Medium (DMEM) Ready Mix, Thermo Fisher Scientific, Waltham, Massachusetts, USA #31966047) supplemented with 10% foetal bovine serum (FBS, Thermo Fisher Scientific #10270106), 100 U/mL penicillin, and 100 µg/mL streptomycin (Thermo Fisher Scientific #15140122) at 37 °C, 5% CO_2_, and 3% (primary HUFs) or 20% O_2_ (immortalised HUFs) adjusted by an incubator with an oxygen sensor and a nitrogen source (Heracell™ 150i). Experiments outside the incubator were performed at atmospheric oxygen levels. For passaging, cells were incubated with 0.05% trypsin–EDTA (Thermo Fisher Scientific #25300054) for 2–10 min and resuspended in growth medium. Cell numbers were determined using an improved Neubauer Haemocytometer according to the manufacturer’s instructions and 16,000 cells/cm^2^ were reseeded into flasks or multiwell plates if not otherwise specified.

### Crystal violet staining assay for cell survival

Cell viability was assessed by crystal violet staining, which stains adherent cells by binding to the DNA and proteins. Dead cells detached from the dish will be washed off prior to staining and, consequently, the staining is indicative of viable cells in the wells. Primary HUFs were seeded into 96-well plates and treated the next day with acrylamide or glycidamide diluted in growth medium at concentrations up to 10 mM (acrylamide) or 2.5 mM (glycidamide) for 24 or 48 h. A third time-point was included for each compound in which the treatment medium was replaced with fresh growth medium after 24 h and cells were allowed to grow for a further 24 h (24 h + 24 h). Treatment was performed in three replicate wells at 37 °C, 5% CO_2_, and 3% O_2_. At 24 and 48 h after treatment, cells were washed with 180 µL PBS followed by staining with 30 µL 0.1% (*w/v*) crystal violet dye (Sigma #C3886) in 10% ethanol (Sigma-Aldrich #32221) for at least 10 min. Cells were washed twice with PBS to remove excess crystal violet and air-dried at room temperature. Crystal violet was solubilised in 100 µL 50% ethanol per well and absorbance was determined at 595 nm using a plate reader. Data are shown as the mean values of absorbance per well relative to control cells and are representative for at least three independent experiments.

### Sample preparation for DNA adduct analysis

Primary HUFs were seeded into 175-cm^2^ flasks and exposed the next day to cytotoxic and sub-cytotoxic concentrations of glycidamide (for 24 h) or acrylamide (for 48 h) diluted in growth medium. Treatment was performed at 37 °C, 5% CO_2_, and 3% O_2_ (*n* = 4). Cells were then harvested and stored as pellets at – 20 °C until DNA was isolated using a standard phenol–chloroform extraction method as described previously (Kucab et al. 2015). DNA concentration and purity of the samples was assessed spectrophotometrically, and 20 µg DNA of each sample was dried using a SpeedVac Concentrator (SVC-100, Savant) for 1.5 h. Three biological replicates were prepared for each treatment condition. Samples were then shipped to the National Center for Toxicological Research (Jefferson, Arkansas, USA) where they were reconstituted in 100 μL water and heated at 100ºC for 15 min to release the N7-GA-Gua adduct from DNA. After cooling to room temperature, each sample was filtered through a 3 kDa filter (Centrifugal Filter Unit, Amicon Ultra–0.5 mL, Ultracel- 3 K, Merck Millipore, Ireland) that had been pre-washed twice with 400 µL water to remove residual glycerine. Filtrates (60 µL) were evaporated to dryness under vacuum and reconstituted in 60 µL acetonitrile:water:formic acid (80:20:0.1, *v/v*) and analysed by UPLC–ESI–MS/MS.

### DNA adduct analysis by UPLC–ESI–MS/MS

The analyses were conducted following the modification of the procedures reported by Gamboa da Costa et al. (2003). Briefly, the analyses were conducted on an Acquity I-class UPLC system coupled to a Xevo TQ-S triple quadrupole mass spectrometer (Waters Corporation, Milford, Massachusetts, USA) operated in positive electrospray ionization mode. MassLynx software, version 4.1, was used for data acquisition and processing. Chromatographic separations were performed on an Acquity UPLC BEH HILIC column (Waters Corporation, 2.1 mm I.D. × 100 mm, particle size 1.7 μm) equipped with a 0.2 μm in-line frit. The column temperature was kept at 30 °C. The mobile phase consisted of mixtures of (A) 10 mM ammonium acetate and (B) acetonitrile. The analyte was eluted with the following gradient: 0–3.60 min, 8% (A); at 4.0 min, 60% (A); 4.0–5.50 min, 60% (A); at 6.00 min return to initial conditions for column equilibration. The flow rate of 0.4 mL/min was kept constant during the 9 min analytical run. The autosampler was maintained at 20 °C. The mass spectrometer source parameters and multiple-reaction monitoring (MRM) method were optimised by infusion of authentic standard of the N7-GA-Gua adduct using flow injection analysis. The optimised parameters were as follows: source temperature 150 °C, desolvation temperature 500 °C, desolvation gas flow 1000 L/h, cone gas flow 150 L/h, collision gas flow 0.15 mL/min, and capillary voltage 0.8 kV. The MRM parameters for the main transition used for semi-quantitation were 239.10 > 152.0, cone voltage 26.0 V, and collision energy 18.0 eV. A secondary transition was used for confirmation (239.10 > 135.0, cone voltage 26.0 V, and collision energy 28.0 eV). The method’s lower limit of quantitation (LLOQ) was determined as 2.5 pg/mL N7-GA-Gua in DNA, which corresponds to 1.7 adducts/10^8^ nucleosides. Because no internal standard (isotope-labelled N7-GA-Gua) was utilised, only semi-quantitation was performed.

### Western blotting

Primary HUFs were seeded into 6-well plates at 270,000 cells/well for 24 h and 150,000 cells/well for 48 h experiments and treated at 37 ºC, 5% CO_2_ and 3% O_2_ the following day with cytotoxic and sub-cytotoxic concentrations of acrylamide or glycidamide. Immortalised HUFs were seeded into 25-cm^2^ flasks at required dilution and treated when they reached 40–50% confluency for 24 h ± 10 μM Nutlin-3a (Cayman Chemicals, Ann Arbor, Michigan, USA #18585) in DMSO. Treated cells were washed with PBS and lysed in 62.5 mM Tris (pH 6.8), 1 mM EDTA (pH 8.0), 2% sodium dodecyl sulphate (SDS), and 10% glycerol supplemented with 1X Halt™ Protease and Phosphatase Inhibitor Cocktail (Thermo Fisher Scientific #78422). The expression of phospho-p53 (Ser15), p53, p21, γ-H2ax (Ser139), phospho-Chk1 (Ser345), and glyceraldehyde 3-phosphate dehydrogenase (Gapdh) was assessed in primary HUFs. In immortalised HUFs, the expression of p53, p21, Mdm2, Gapdh, and β-actin was evaluated. Western blotting was performed as described previously (Wohak et al. 2016) using the following antibodies: anti-phospho-p53 (1:2,000; Cell Signalling, Danvers, Massachusetts, USA #9284), anti-p53 (1:500; Cell Signalling, 2524S), anti-p21 (1:2,000; BD Biosciences, Franklin Lakes, New Jersey, USA #BD556431), anti-γ-H2ax (1:1,000; Cell Signalling #9718), anti-phospho-Chk1 (1:1,000; Cell Signalling #2348), anti-Mdm2 (1:750; Abcam, Cambridge, United Kingdom #ab16895), Gapdh 1:25,000; Chemicon International, Temecula, California, USA #MAB374), and anti-β-actin (1:25,000; Abcam #ab6276).

### *lacZ* mutation assay

The *lacZ* mutation assay is a reporter gene assay that provides a relatively quick estimate of mutagenicity in primary HUFs prior to the HIMA and *lacZ* mutagenicity has been shown to be a good predictor of *TP53* mutagenicity (Kucab et al. 2016). For the *lacZ* assay, primary HUFs were seeded into 75-cm^2^ flasks (five per treatment) and treated the following day with cytotoxic and sub-cytotoxic concentrations of glycidamide or acrylamide, or with solvent control (water). To align the time-points for both compounds, growth medium on glycidamide-treated cells was replaced by fresh growth medium after 24 h for a further 24 h. At 48 h post-treatment, cells were counted and seeded at 2 × 10^6^ cells into 175-cm^2^ flasks to allow cells to proliferate and fix mutations. After approximately six cell doublings (4–5 days), cells were harvested, and pellets stored at – 20 ºC until DNA isolation using a standard phenol–chloroform extraction as described previously (Kucab et al. 2015). pUR288 (*lacZ*) plasmid rescue and mutant frequency were determined as described previously (Kucab et al. 2016; Mahabir et al. 2009). Briefly, 30 μg DNA were digested with HindIII and incubated with magnetic beads coated with lacI fusion protein. After plasmids were eluted from the beads using isopropyl-β-D-thiogalactopyranoside (IPTG), they were circularised at the HindIII sites using T4 DNA ligase. Circularised plasmids were electroporated into *Escherichia coli* lacking β-galactosidase (*lacZ*^*−*^) and galactose epimerase (*galE*^*−*^). To select for mutants, one thousandth of the transformed bacteria were plated on non-selective, titre plates containing 5-bromo-4-chloro-3-indolyl-D-galactopyranoside (X-Gal), and the remainder on mutant selective plates containing the lactose analogue phenyl-β-D galactosidase (P-Gal). Only bacteria harbouring a mutation in the *lacZ* plasmid can grow on the selective plates, while bacteria containing the non-mutated, WT *lacZ*^+^ plasmid will undergo lysis. Mutant frequency was determined as the ratio between the number of mutant colonies on the selective plates to the number of colonies formed on the non-selective titre plate (× dilution factor 1000).

### Gene expression analysis by qRT-PCR

Primary HUFs were seeded into 25-cm^2^ flasks and incubated overnight. The acrylamide-containing medium was added, and cells incubated for a further 48 h, after which RNA was extracted from cell pellets using the RNeasy Mini Kit (QIAGEN, Hilden, Germany #74104) according to the manufacturer’s instructions. After establishing the concentration and quality parameters of the RNA using a NanoDrop spectrophotometer, RNA was reverse transcribed into cDNA using a High-Capacity RNA-to-cDNA™ Kit (Thermo Fisher Scientific #4387406). qRT-PCR was performed using a 2X TaqMan™ Gene Expression Master Mix (Thermo Scientific #4369016) and the Roche Universal Probe Library designed intron-spanning assay (i.e., primers and matching probe) for the *Cyp2e1* NCBI sequence NM_021282.2. Gene expression was analysed using a 7500 Fast Real-Time PCR System (Applied Biosystems). Relative gene expression was normalised to the housekeeping gene *Gapdh* (NM_001289726.1) and analysed by the comparative threshold cycle (*C*_t_) method. Results were reported as the fold change in gene expression between the treated and untreated (control) samples (2^−ΔΔCt^ method).

### HUF immortalisation assay (HIMA)

The HIMA was performed to create immortalised HUF clones. The principle of the HIMA relies on the fact that primary mouse embryo fibroblasts (MEFs) undergo senescence after ~ 2 weeks when cultured at atmospheric oxygen conditions. Due to mutations in genes related to senescence, they can start proliferating again and become immortalised cell lines. MEFs, in contrast to human cells, can be immortalised by mutations in *TP53* alone, making it possible to generate and select *TP53*-mutant HUFs. A detailed assay protocol has been published previously (Hölzl-Armstrong et al. 2019). In brief, primary HUFs were seeded into Corning^®^ CellBIND^®^ 6-well plates and treated the next day with 1.1 mM glycidamide (*n* = 198), 1.5 mM acrylamide (*n* = 24), 3 mM acrylamide (*n* = 6), or solvent control (water, *n* = 30). After 24 h, the treatment medium was replaced with growth medium on the glycidamide- and solvent control-treated cultures. Once the cells reached confluence, they were sub-cultured at 1:2–1:5 on Corning^®^ CellBIND^®^ 6-well plates. Five days post-treatment, cells were moved to atmospheric oxygen levels to induce senescence crisis and, consequently, selected for senescence bypass. Cultures were serially passaged at dilutions of 1:1.5–1:4 until senescence crisis, during which growth medium was changed at least every 3 days. As soon as cells started to divide again (i.e., immortalised clones emerged), serial passaging was continued. Cultures with no obvious clones emerging were passaged at least every 14 days by passaging all cells to a new well. Cultures were split continuously for some passages at dilutions of 1:2–1:5. Once the culture appeared homogenous and was able to repopulate a well on a 6-well dish within 6 days after being split 1:3–1:50, a Nutlin-3a counter-screen was performed. WT-*TP53* HUFs are sensitive towards Nutlin-3a treatment, which means that p53 is activated and cells stop growing after treatment with Nutlin-3a. In contrast, HUFs with mutations in *TP53* are resistant to Nutlin-3a and continue to grow in its presence. In addition, mixed HUF cultures showing both sensitive *TP53*-WT and resistant *TP53*-mutant cells are possible. A more detailed description of the Nutlin-3a counter-screen can be found elsewhere (Hölzl-Armstrong et al. 2019; Kucab et al. 2017). For the Nutlin-3a, counter-screen cultures were split into two wells of a 6-well plate at the desired dilution depending on the growth rate of the clone (1:3–1:50). The following day one well was treated with 10 µM Nutlin-3a in DMSO, while the other well remained untreated. Cultures were visually inspected under a microscope after 5 days to determine if cultures were resistant, sensitive, or showed a mixed response to Nutlin-3a treatment. Mixed-response and resistant cultures were expanded to larger flasks (25, 75 cm^2^), and frozen stocks and pellets were prepared. Whole-cell lysates after 24 h treatment with Nutlin-3a were collected to investigate the induction of p53-pathway proteins (p53, p21, Mdm2) by Western blotting.

### *TP53* mutation analysis

DNA was extracted using the Gentra Puregene Cell Kit B (Qiagen #158745) according to the manufacturer’s instructions. PCR was performed to amplify exons 4–9 (including introns) of human *TP53*. From cultures without identified mutation in exons 4–9, but clear resistant or mixed responses in the Nutlin-3a counter-screen, RNA was extracted from pellets and reverse transcribed to cDNA as described above. DNA and cDNA were amplified using primers and cycling conditions as described by Hölzl-Armstrong et al. (2019). Amplification products were run on a 2% agarose gel (Thermo Fisher Scientific #15310–019) containing 0.5 µg/mL ethidium bromide (Sigma-Aldrich #E1510). Band size and concentration of each product were estimated against 6 µL of a 100 base pair DNA ladder (Qiagen #239045) loaded on each gel. The amplification products were then submitted to GENEWIZ (Takely, UK) for purification and Sanger dideoxy sequencing using the sequencing primers described previously (Hölzl-Armstrong et al. 2019). The freely available software Chromas (Technelysium Pty Ltd, Australia) was used to visually inspect the sequences and to export the FASTA sequence. Those were aligned against the human *TP53* reference sequence NC_000017.11 from GenBank using the Basic Local Alignment Search Tool for Nucleotides (BLASTN) from the National Center for Biotechnology Information (NCBI) (https://blast.ncbi.nlm.nih.gov/Blast.cgi). The mutation feature search tool of the IARC TP53 mutation database (https://p53.iarc.fr/TP53GeneVariations.aspx) was used to assess gene variations. Mutations that were found using BLASTN were visually inspected in the chromatogram and classified as homo-/hemi- or heterozygous. Mutations were confirmed by sequencing DNA from an independent sample of cells from the same culture.

### Preparation of single-cell clones for WGS analysis

Selected clonal cell lines established in the HIMA were thawed into 75-cm^2^ flasks and placed in an incubator. Once cultures were confluent each was seeded at a limiting dilution of 5 cells/mL into two 96-well plates (100 µL/well). To ensure that each clone had arisen from a single cell, wells were visually inspected daily. Wells in which a big area was populated too quickly after seeding were not considered to ensure that the clone really had come from a single cell. The medium was changed every 3–4 days. Once at least half of the well was populated by the clone, all cells were moved to a 24-well plate, a 6-well plate, a 25-cm^2^ flask, and finally a 75-cm^2^ flask from which pellets were prepared. DNA of single-cell clones and selected primary HUFs was extracted using a standard phenol–chloroform method. The purity and concentration of the DNA was established using a NanoDrop spectrophotometer and the samples diluted to 20 ng/μL in TE-buffer.

### DNA quality control and quantification and library preparation

Samples were quantified using the fluorescence-based AccuClear^®^ Ultra High-sensitivity dsDNA kit (Biotium, Fremont, California, USA #31028). In brief, 200 nL of sample DNA was added in triplicate along with the AccuClear^®^ Ultra High-sensitivity DNA standards into a black Nunc^®^ MaxiSorp™ 384-well assay plate (Thermo Scientific # 460518) using a Mosquito LV liquid-handling platform. Using a Bravo (384ST head) liquid-handling platform, fluorescent AccuClear^®^ buffered dye was added to each well. To obtain the concentration and quality of each sample, the plate was read on a BMG LabTech FLUOstar Omega plate reader. Next, samples were cherrypicked to 200 ng/120 µL using a Tecan liquid-handling platform and sheared to 450 bp using a Covaris LE220 instrument. Post-sheared samples were then purified using Agencourt^®^ AMPure^®^ XP SPRI beads (Beckman Coulter Inc., Brea, California, USA #A63880) on an Agilent Bravo WS automation system. Library preparation (end-repair, A-tailing and ligation) was performed with the NEBNext^®^ Ultra™ II custom kit (New England Biolabs #E7645L) on an Agilent Bravo WS automation system. PCR was set up using KAPA HiFi HotStart ReadyMix (Roche # KK2601) and 96 iPCR tag barcodes (Integrated DNA Technologies Inc., Coralville, Iowa, USA) on an Agilent Bravo WS automation system. Samples were amplified by running the PCR programme shown in Supporting Table S1. Next, amplified samples were purified using Agencourt^®^ AMPure^®^ XP SPRI beads on a Beckman BioMek NX-96 liquid-handling platform and quantified with the AccuClear^®^ Ultra High-sensitivity dsDNA kit using Mosquito LV liquid-handling platform, Bravo WS, and BMG FLUOstar Omega plate reader as described above. After pooling the libraries in equimolar amounts on a Beckman BioMek NX-8 liquid-handling platform, they were normalised to 2.8 nM ready for cluster generation on a c-BOT and loading on Illumina sequencing platform.

### WGS and processing of WGS data

WGS was performed on an Illumina XTEN^®^ machine as per the operator’s instructions. Acquired sequences were analysed for quality and finally aligned to the murine reference genome (Mus musculus GRCm38 and ensembl 84 transcriptome) using the alignment software Burrows-Wheeler Aligner (BWA, versions VN:0.7.16a-r1181 and VN:0.7.17-r1188). SBS and indel (ID) mutations were discovered using the cancer variants through expectation maximization (CaVEMan; https://cancerit.github.io/CaVEMan/) and Pindel (https://cancerit.github.io/cgpPindel/) algorithms utilising the closest primary HUF as the matched normal. The CaVEMan algorithm employs a naïve Bayesian classifier to obtain the probability of all possible mutations at each nucleotide. The Pindel algorithm can detect medium-sized insertions, large deletions, tandem duplications, and other structural variants using a pattern growth approach by identifying the breakpoints of these variants (Ye et al. 2009). Mutations were further filtered by Pindel (PASS) filters and the CaVEMan filters for the clipping index (CLPM) and alignment score median (ASRD), which were set to default CaVEMan settings (CLPM = 0; ASRD ≥ 0.93). In addition, SBS variants with a variant allele frequency (VAF) ≤ 0.2 were excluded.

### Extraction of mutational signatures

The SigProfilerExtractor tool was used to extract mutational signatures (Version 0.0.5.77, https://pypi.org/project/sigproextractor/). SigProfilerExtractor uses multiple extractions of signatures by non-negative matrix factorisation (NMF) with various random initial conditions as described previously (Alexandrov et al. 2020). The extracted signatures were further normalised to the human genome trinucleotide frequency and compared with known COSMIC signatures (V3, May 2019) and relevant other signatures from literature. Cosine similarity was calculated to allow comparison. A cosine similarity of 1.0 reflects a perfect match.

### Statistics

Results are shown as mean values ± standard deviation (SD). The sample size is indicated in each section. Statistical analysis was performed using *GraphPad Prism* version 8.2.0 (GraphPad Software Inc., La Jolla, CA, USA). Groups of two were compared by two-sample *t* test assuming that unequal variances and groups of three or more were compared by one-way analysis of variance (ANOVA). Statistical analysis of the relative mRNA expression was performed by log2 transforming the data and analysing it using a single-sample *t* test with Bonferroni correction against the population control mean of 0. Significance levels are * *p* < 0.05, ** *p* < 0.01, and *** *p* < 0.001.

## Results

### Cell viability in primary HUFs after treatment with acrylamide and glycidamide

**Fig. 1:**
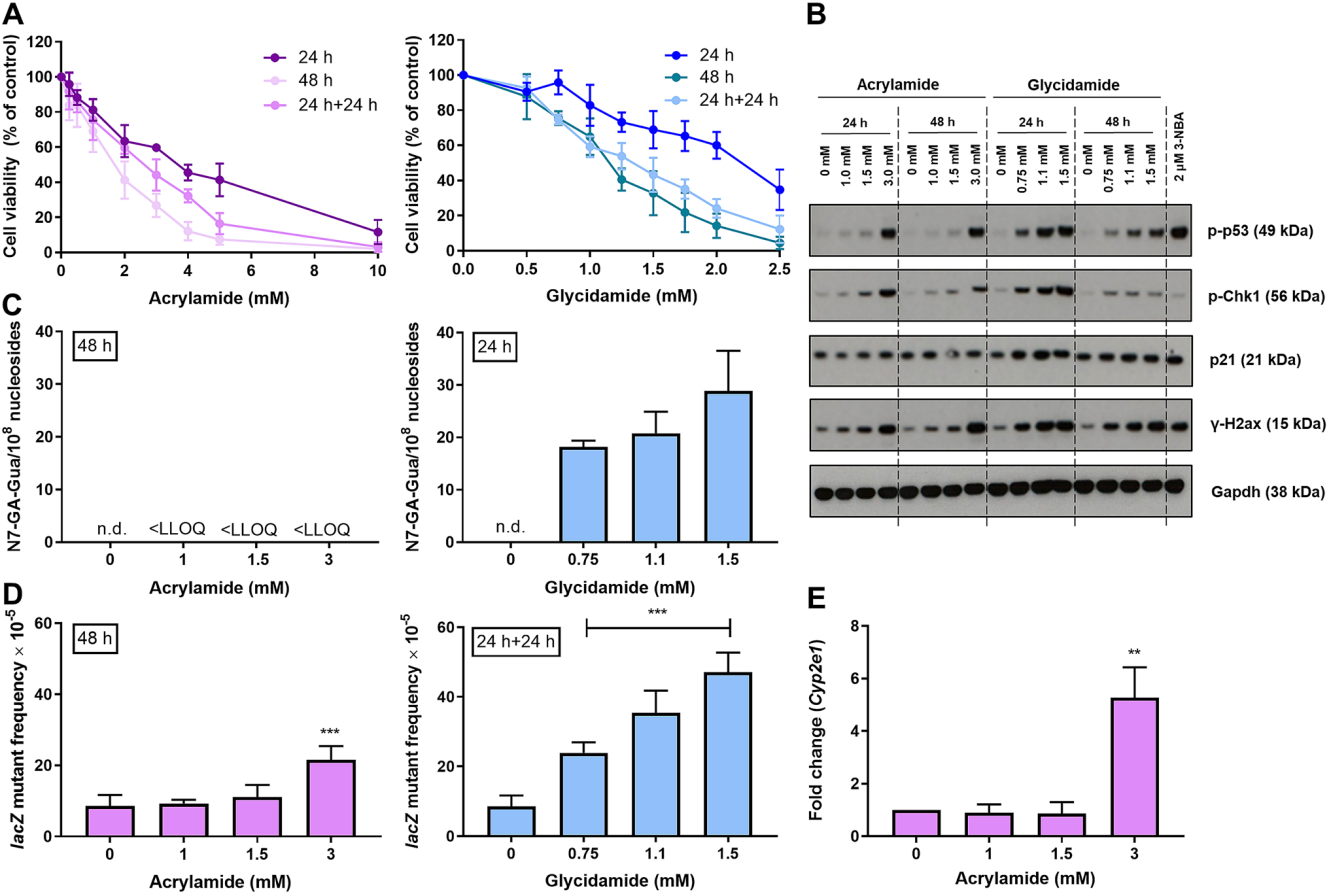
Optimisation of treatment conditions for the HIMA. **a** Cell viability assessment: Primary HUFs were treated with indicated concentrations of acrylamide (left panel) or glycidamide (right panel) for up to 48 h, and cell viability (% control) was assessed by staining with crystal violet. Cells treated with water served as controls. Shown are mean values ± SD (n > 3). **b** Western blot analysis of various DDR proteins (p-p53, p-Chk1, p21 and γ-H2ax) in primary HUFs exposed to indicated concentrations of acrylamide or glycidamide for 24 or 48 h. Gapdh was used as a loading control and 3-NBA (2 µM, 48 h) included as positive control. Representative images of Western blot analysis are shown. Analysis was performed in duplicate from independent experiments. **c** DNA adduct analysis. Primary HUFs were treated as indicated with acrylamide (left panel) or glycidamide (right panel) for 48 or 24 h, respectively, and the formation of N7-GA-Gua adducts was quantified by UPLC-ESI–MS/MS analysis. Cells treated with water served as controls. Shown are mean values ± SD (*n* > 3). LLOQ, lower limit of quantification; n.d., not detected. **d** Induction of *lacZ* mutants in primary HUFs treated with acrylamide (48 h; left panel) or glycidamide (24 h + 24 h; right panel). Cells treated with water served as controls. After a total of 48 h, cells were passaged to a new flask and allowed to double six times to fix DNA mutations. *LacZ* mutant frequencies were calculated as the number of mutant colonies per number of recovered transformants. Shown are mean values ± SD (*n* > 3). Statistical analysis was performed by one-way ANOVA followed by Dunnett’s test for multiple comparison values (*** *p* < 0.001 compared with control). **e** Relative gene expression of *Cyp2e1* in primary HUFs treated with acrylamide for 48 h. *Cyp2e1* expression was determined by qRT-PCR and the 2^−ΔΔCt^ method. All values are normalised to mRNA expression of the housekeeping gene *Gapdh* and are relative to the water control (*n* > 3). Statistical analysis was performed by log2 transforming the data and analysing it using a single-sample *t* test with Bonferroni correction against the population control mean of 0 (** *p* < 0.01)

Cell viability in primary HUFs after treatment with various concentrations of acrylamide (0–10 mM) or glycidamide (0–2.5 mM) for 24 or 48 h was assessed using crystal violet staining. A recovery period of 24 h in fresh medium was also included after the 24 h treatment. All exposures led to a concentration-dependent decrease of cell viability, with glycidamide being more cytotoxic than acrylamide (Fig. 1a). Both compounds also exhibited a significant time-dependent decrease in viability when comparing the 24-h exposure with the 48-h and 24-h + 24-h time-points (*p* < 0.05). In contrast, no difference was found between the 24-h + 24-h and 48-h treatment (*p* > 0.05). Based on the cell viability results, three concentrations per compound were selected for subsequent experiments. As cell numbers for the HIMA are optimised for a 48-h treatment, concentrations for acrylamide were based on the 48-h exposure period results, because it must be metabolised to exert its genotoxic properties, while for glycidamide, which does not require metabolic activation, the 24-h exposure period was chosen. To align the time-points, concentrations selected were based on the 48 h (acrylamide) and 24-h + 24-h (glycidamide) experiments and as follows: 1, 1.5, and 3 mM for acrylamide and 0.75, 1.1, and 1.5 mM for glycidamide. For each compound, the three concentrations selected lead to 80–60, 60–40, and 20–40% cell viability in treated HUFs relative to control, respectively.

### Induction of DNA damage response proteins in primary HUFs after treatment with acrylamide and glycidamide

Induction of the DDR markers p-p53, p-Chk1, p21, and γ-H2ax was assessed in primary HUFs after treatment with the selected acrylamide and glycidamide concentrations for 24 and 48 h by Western blotting (Fig. 1b). All DDR proteins except p21 were induced by acrylamide after exposure for 24 h. An extension of the treatment time to 48 h led to a response very similar to that at 24 h for all DDR markers except p-Chk1, which was induced to a lesser extent after 48 h compared to 24 h. Treatment with glycidamide for 24 h induced all DDR proteins and their induction decreased after 48 h. Generally, p21 was highly expressed at all glycidamide concentrations.

### N7-GA-Gua formation in primary HUFs after treatment with acrylamide and glycidamide

N7-GA-Gua adduct formation was analysed after treatment of primary HUFs with acrylamide and glycidamide using UPLC-ESI–MS/MS (Gamboa da Costa et al. 2003; Segerback et al. 1995) to demonstrate the ability of both compounds to result in DNA adducts, and thus induce pre-mutagenic DNA lesions. A representative set of adduct chromatograms for N7-GA-Gua obtained from primary HUFs treated with water, 1.5 mM acrylamide, or 1.5 mM glycidamide is shown in Supporting Fig. S2.

Consistent with the other studies in mammalian cells (Martins et al. 2007; Mei et al. 2008b), including primary HUFs (Zhivagui et al. 2019), acrylamide did not form any quantifiable amounts of N7-GA-Gua adducts under the experimental conditions used, while treatment with glycidamide resulted in a concentration-dependent formation of N7-GA-Gua (Fig. 1c). Glycidamide treatment with both 0.75 and 1.1 mM led to ~ 20 adducts/10^8^ nucleosides, with a higher level of adduct formation, ~ 30 adducts/10^8^ nucleosides, after exposure to 1.5 mM glycidamide.

### Induction of *lacZ* mutants in primary HUFs after treatment with acrylamide and glycidamide

Induction of *lacZ* mutants after exposure to acrylamide and glycidamide was analysed in primary HUFs to guide the selection of suitable treatment conditions for the HIMA. *LacZ* mutants were induced in a concentration-dependent manner by both compounds (Fig. 1d). Treatment with solvent control (water) led to a background mutant frequency of ~ 9 × 10^−5^. Treatment with acrylamide induced a significant increase in *lacZ* mutants only at the highest concentration tested (i.e., 3 mM), where the mutant frequency, at ~ 22 × 10^−5^, was twice the background frequency. In agreement with the DNA adduct data, a concentration-dependent increase in *lacZ* mutant frequency was observed for glycidamide. While with 0.75 mM glycidamide, the mutant frequency was already doubled to ~ 22 × 10^−5^, and treatment with 1.1 mM further increased the mutant frequency to ~ 35 × 10^–5^. At the highest glycidamide concentration tested (i.e., 1.5 mM), the mutant frequency was ~ 45 × 10^−5^. Comparison of acrylamide and glycidamide at concentrations that caused similar levels of cytotoxicity showed that glycidamide-induced three times more *lacZ* mutants than acrylamide (e.g., 1.5 mM acrylamide vs. 1.1 mM glycidamide: ~ 11 × 10^−5^ vs. ~ 35 × 10^−5^).

### *Cyp2e1* gene expression in primary HUFs after treatment with acrylamide

As acrylamide is activated to glycidamide mainly by Cyp2e1, expression of this gene after treatment with acrylamide was quantified by qRT-PCR in primary HUFs. As seen in Fig. 1e, the expression of *Cyp2e1* was generally very low, but it increased ~ sixfold after treatment with 3 mM acrylamide, but not at lower concentrations. This correlates with *lacZ* mutagenicity, where an increase of mutant frequency occurred only at this concentration.

### *TP53* mutations induced by glycidamide in immortalised HUFs

#### Choice of treatment conditions and number of cultures for the HIMA

Because the highest induction levels of DDR proteins were observed after 24-h exposure to glycidamide, it was decided to treat primary HUFs for 24 h to initiate the HIMA. To ensure that enough cells were viable and able to proliferate after treatment, cells were exposed to 1.1 mM glycidamide to achieve a cell survival of 40–60%. For acrylamide, which, in contrast to glycidamide, needs to be metabolically activated, HUFs were treated for 48 h to initiate the HIMA. To ensure similar levels of cell viability for the HIMA as observed with glycidamide, a concentration of 1.5 mM acrylamide was selected. The HIMA with glycidamide was performed using 198 cultures over a total of three separate immortalisation assays, while, for acrylamide, one HIMA with 24 cultures was performed. In addition, as *lacZ* mutants and *Cyp2e1* mRNA expression were induced only after exposure to 3 mM acrylamide, six HUF cultures were treated with this concentration to generate immortalised HUFs for WGS analysis. Thirty control cultures were treated with water and passaged alongside the carcinogen-treated cultures to serve as controls.

#### Nutlin-3a counter-screen and sequence analysis of immortalised HUFs

Using the Nutlin-3a counter-screen, 11% (21/198) of HUFs immortalised after glycidamide treatment were found to be *TP53* mutants, while none (0%) of spontaneously immortalised and acrylamide-treated cultures were *TP53* mutants (Table 1). Human exons 4–9 including adjacent introns of *TP53*-mutant cultures were amplified using PCR and subjected to Sanger sequencing. Although cultures GA-52, -116, -126, -134, and -194 were resistant to Nutlin-3a treatment, no *TP53* mutations were identified in exons 4–9 of cultures GA-52, -116, -126, and -134, while exon 7 of culture GA-194 could not be amplified or sequenced. Thus, culture GA-194 was classified as a “complex” mutation (further discussed below). The overall 11% for glycidamide-treated HUFs based on the Nutlin-3a counter-screen sub-divided into 9% for mutations in the human *TP53* sequences and 2% for mutations within exons of the murine *Trp53* gene.

**Table 1:** Overview of treatment conditions, *TP53* mutation frequency, and pattern detected in glycidamide- and acrylamide-treated as well as spontaneously immortalised (i.e., controls) HUFs

	Glycidamide	Acrylamide		Control
Treatment concentration	1.1 mM	1.5 mM	3 mM	water
Treatment time	24 h	48 h		24 h
Total HUF cultures	198	24	6	30
*TP53*-mutant immortalised clones	21	0		0
Frequency of all *TP53*- and *Trp53*-mutant clones	11% (21/198)	0% (0/30)		0% (0/30)
Frequency within human exons	9% (17/198)	0% (0/0)		0% (0/0)
Frequency within mouse exons	2% (4/198)	0% (0/0)		0% (0/0)
Mutation type				
G > T/C > A	6% (1/17)	0% (0/0)		0% (0/0)
G > C/C > G	18% (3/17)	0% (0/0)		0% (0/0)
G > A/C > T	12% (2/17)	0% (0/0)		0% (0/0)
A > T/T > A	35% (6/17)	0% (0/0)		0% (0/0)
A > G/T > C	18% (3/17)	0% (0/0)		0% (0/0)
A > C/T > G	6% (1/17)	0% (0/0)		0% (0/0)
Complex	6% (1/17)	0% (0/0)		0% (0/0)
Mutations on transcribed strand	25% (4/16)	0% (0/0)		0% (0/0)
Mutations on non-transcribed strand	75% (12/16)	0% (0/0)		0% (0/0)

Mutations were detected by Sanger dideoxy sequencing. Brackets show the number of *TP53*-mutant clones versus the total clones analysed

#### Mutation pattern induced by glycidamide in the human *TP53* gene in immortalised HUFs

An overview of the mutation pattern in the human *TP53* gene is shown in Table 1. The predominant mutation type induced by glycidamide in human *TP53* exons 4–9 was A > T/T > A transversions (35%), followed by 18% each A > G/T > C and G > C/C > G mutations. Furthermore, 12% G > A/C > T and 6% of each A > C/T > G transitions, G > T/C > A transversions, and complex mutations were found. Overall, 59% of *TP53* mutations were identified at A:T and 36% at G:C base pairs with only one mutation harboured at a CpG site. Most *TP53* mutations (75%) were found on the non-transcribed strand indicating a transcription bias.

#### Exon and codon distribution of glycidamide-induced *TP53* mutations in immortalised HUFs

A summary of each *TP53* mutation and its characteristics can be found in Table 2. All identified human *TP53* mutations but one (GA-194) were SBS that occurred either in the coding sequence or the splice sites of the sequenced human exons 4–9 and resulted in amino acid substitutions. Furthermore, each *TP53*-mutant harboured only one mutation and no indels were induced. Most *TP53* mutations were found in exons 5 and 8 (6/17 each). While no mutations were identified within exon 4 or 9, 2/17 were harboured within each exon 6 and 7. Interestingly, only one of six hotspot codons was targeted in this assay (G245 in GA-196). GA-196 was also the only *TP53* mutation found at a residue that is crucial for the structure of p53, while two mutations (GA-44 and -48) were found in one (R280) of the seven residues that make direct contact with the DNA. Furthermore, mutation GA-29 (C176) was harboured in a region involved in zinc binding. Three residues, namely C135 (GA-39, -40), E280 (GA-44, -48), and E286 (GA-13, 118), were targeted at the same base twice.

**Table 2:** Summary of *TP53* mutations in immortalised HUFs induced by glycidamide

GA-	Nutlin-3a response	Exon	Codon^a^	Mutation type	Strand	WT	MUT	Coding change	Zygosity	Activity	Effect
5	Resistant	6	220	A > G/T > C	NTS	TAT	TGT	Y220C	Homo-/hemi-	NF	Missense
13	Mixed	8	286	A > T/T > A	NTS	GAA	GAT	E286D	Hetero-	NF	Missense
23	Resistant	–	in. 6 (SA)	A > T/T > A	NTS	AG	TG	splice	Homo-/hemi-	NA	Splice
29	Resistant	5	176	G > T/C > A	NTS	TGC	TTC	C176F	Hetero-	PF	Missense
39	Resistant	5	135	G > C/C > G	TS	TGC	TGG	C135W	Homo-/hemi-	PF	Missense
40	Resistant	5	135	G > C/C > G	TS	TGC	TGG	C135W	Homo-/hemi-	PF	Missense
44	Resistant	8	280	A > T/T > A	NTS	AGA	TGA	R280^a^	Homo-/hemi-	NA	Nonsense
48	Resistant	8	280	A > T/T > A	NTS	AGA	TGA	R280^a^	Homo-/hemi-	NA	Nonsense
77	Resistant	5	168	A > T/T > A	NTS	CAC	CTC	H168L	Homo-/hemi-	NF	Missense
82	Mixed	6	205	A > G/T > C	NTS	TAT	TGT	Y205C	Hetero-	NF	Missense
91	Resistant	8	270	A > G/T > C	TS	TTT	TCT	F270S	Homo-/hemi-	NF	Missense
118	Resistant	8	286	A > T/T > A	NTS	GAA	GAT	E286D	Hetero-	NF	Missense
162	Resistant	5	132	A > C/T > G	NTS	AAG	ACG	K132T	Homo-/hemi-	NF	Missense
164	Mixed	5	141	G > C/C > G	TS	TGC	TGG	C141W	Hetero-	NF	Missense
194	Resistant	7	–	–	–	–	–	–	–	–	–
196	Resistant	7	245	G > A/C > T	NTS	GGC	AGC	G245S	Homo-/hemi-	NF	Missense
197	Resistant	8	272	G > A/C > T	NTS	GTG	ATG	V272M	Homo-/hemi-	NF	Missense

Mutations were detected by Sanger dideoxy sequencing and mutation data acquired from the IARC TP53 mutation database (R20, July 2019). Nutlin-3a response was to 10 µM Nutlin-3a treatment for 5 days. Activity refers to the activity of the respective mutation found in the yeast promotor assay according to Kato et al. (2003)

*NF* non-functional, *PF* partially functional, *NA* not analysed, *SA* splice acceptor site, *NTS* non-transcribed strand, *TS* transcribed strand, *MUT* mutant

^a^ Hotspot codons marked in bold

#### Zygosity, Nutlin-3a response, and p53 functionality of glycidamide-induced *TP53* mutants

Mutations in *TP53* can be classified as heterozygous (one mutated allele, one wild-type [WT] allele) or homo-/hemizygous mutations. However, it is not possible to distinguish between homozygous (two alleles with the same mutation) and hemizygous (one mutated, loss of heterozygosity of the second allele) mutations. Most *TP53* mutations (11/17) induced by treatment with glycidamide in immortalised HUFs were homo-/hemizygous (cultures GA-5, -23, -39, -40, -44, -48, -77, -91, -162, -196, -197) with five cultures harbouring heterozygous mutations (GA-13, -29, -82, -118, -164). Three of the heterozygous *TP53* mutations showed a mixed (cultures GA-13, -82, -164) and two resistant (cultures GA-29, 118) responses towards Nutlin-3a. Sequencing of the Nutlin-3a-treated cultures GA-13 N, -82 N, and -164 N revealed that both GA-13 N and -194 N harboured a homozygous *TP53* mutation, which most likely means that the WT allele retained by the untreated culture was lost in the Nutlin-3a-treated cells. The remaining Nutlin-3a-treated cultures still harboured heterozygous *TP53* mutations. Most of the induced *TP53* mutations have been identified as non-functional (10/17) or partially functional (3/17) in the yeast promotor transactivation assay (Kato et al. 2003); no data were available for four mutations. Cultures GA-29 and -196 both exerted a dominant negative effect over WT p53 protein, whereas cultures GA-5, -82, -197 showed only a moderate dominant negative effect. While most of the *TP53* mutations were missense mutations (13/17), two nonsense mutations (cultures GA-44 and -48) were also found. None of the *TP53*-mutant clones harboured a silent mutation.

#### Glycidamide-treated cultures with unidentified* TP53* mutation in immortalised HUFs

In total, 21 out of 198 cultures were either resistant or showed a mixed response towards Nutlin-3a and, thus, were classified as *TP53* mutants. However, four mutants (GA-52, -116, -126, -134) did not harbour a *TP53* mutation in human exons 4–9. Furthermore, one culture had a complex deletion in exon 7 (GA-194), which made it impossible to amplify and sequence exon 7 and, subsequently, identify the deletion. These mutants were treated with a range of Nutlin-3a concentrations (0–10 μM) for 5 days and then stained by crystal violet to assess cell viability (Supporting Fig. S3). Generally, cell viability of *TP53*-WT cells decreased in a concentration-dependent manner after Nutlin-3a treatment with about 15% viable cells left after exposure to 10 µM Nutlin-3a for 5 days (Supporting Fig. S3a). As seen in Supporting Fig. S3e, in comparison with the untreated cells, growth of *TP53*-WT cells was strongly inhibited with hardly any cells remaining after Nutlin-3a exposure. In contrast, the growth of *TP53* mutants was unaffected by Nutlin-3a treatment with more than 80% viable cells remaining after exposure to 10 µM Nutlin-3a for 5 days (see Supporting Fig. S3a and d). These observations are consistent with the previous studies (Kucab et al. 2017). A highly similar cell viability curve to that of a *TP53-*mutant was observed for cultures GA-52, -134, and -194 with cell viability remaining around 80% compared with untreated cells after exposure to 10 μM Nutlin-3a for 5 days (Supporting Fig. S3b). Images of cultures GA-52, -134, and -194 at the end of the Nutlin-3a counter-screen confirmed that these were, indeed, Nutlin-3a resistant, with the morphology of treated cells being indistinguishable from untreated cells (Supporting Fig. S3f–h). Cultures GA-116 and GA-126 showed a concentration-dependent decrease of cell viability in response to Nutlin-3a treatment that was very similar to the typical response of *TP53*-WT clones (Supporting Fig. S3c). After exposure of cells to 10 μM Nutlin-3a, only 20–40% viable cells survived compared with untreated cells. However, after re-exposing the population of cells that survived Nutlin-3a treatment (GA-116 N and GA126N) to Nutlin-3a, cultures exhibited resistance. Images taken at the end of the HIMA Nutlin-3a counter-screen revealed a difference in untreated and treated wells, with Nutlin-3a clearly impacting cell growth (Supporting Fig. S3i and j). However, parts of the well for cultures GA-116 and -126 were populated with viable cells, confirming the mixed-response classification for these clones.

Moreover, cDNA of the five cultures was prepared, exons 2–11 amplified and subjected to Sanger dideoxy sequencing. It was possible to identify mutations in exon 10 of the mouse *Trp53* gene for cultures GA-52, -116, -126, and -134. Interestingly, all *Trp53* mutations were harboured on the transcribed strand and were of the G > T/C > A mutation type. Culture GA-194 was also subjected to cDNA sequencing, but it was only possible to align exons 2–6 and exons 10–11 with the reference sequence of *Trp53*, which further indicates that this culture harbours a complex deletion in exon 7 of *TP53*. Overall, it was surprising that 4/21 (19%) *p53* mutations were outside human exons 4–9 as, in the IARC TP53 database, ~ 90% of the recorded mutations are harboured within exons 5–8 of *TP53* and it seems that, to date, no mutations have been reported to occur outside exons 4–9 in immortalised HUFs.

#### Expression and induction of p53-pathway proteins in glycidamide-treated *TP53* mutants after treatment with Nutlin-3a

**Fig. 2:**
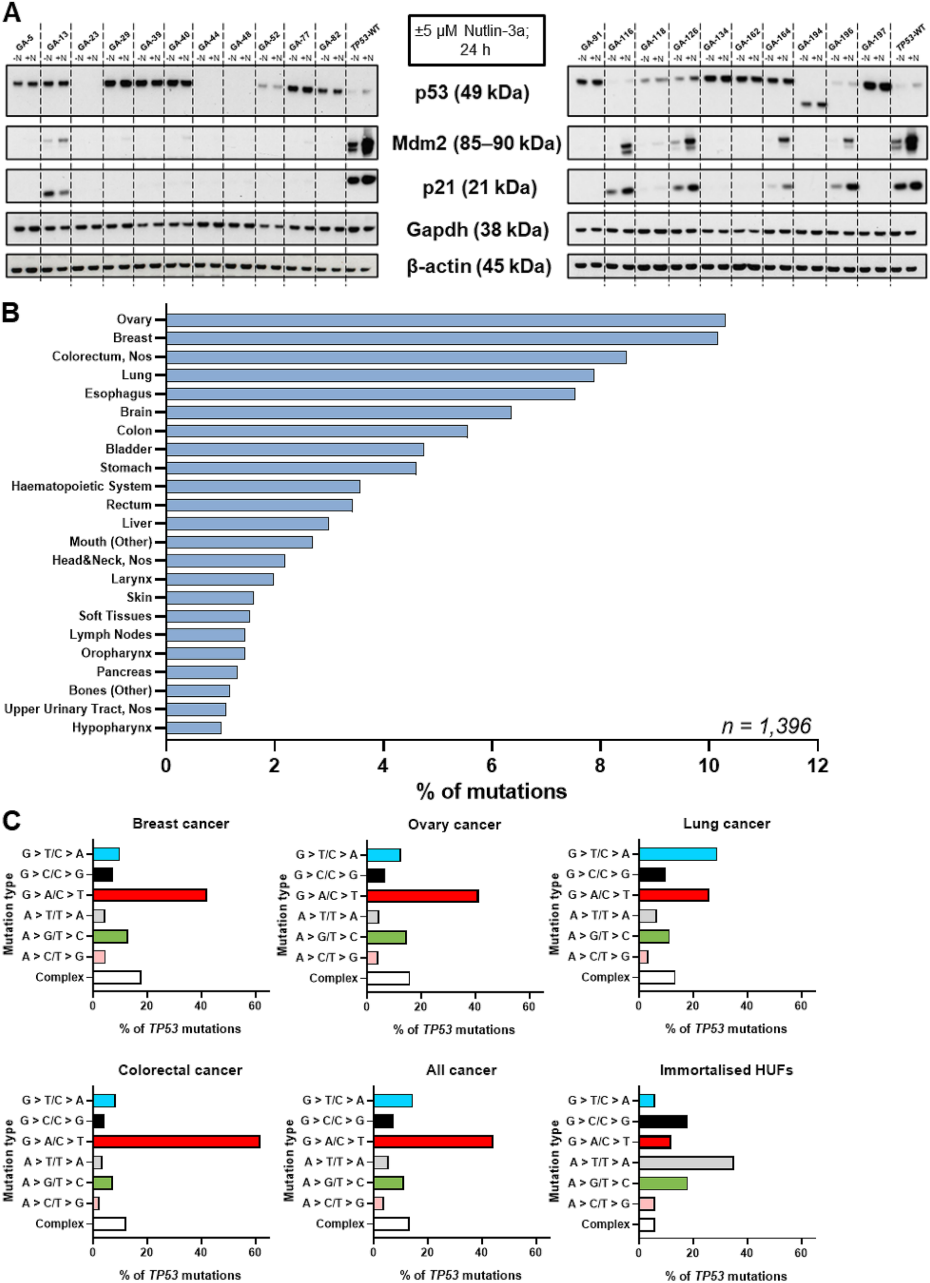
Results of the HIMA. **a** Expression and induction of p53-pathway proteins (p53, p21, Mdm2) after treatment with ( +) or without (−) 10 µM Nutlin-3a for 24 h in glycidamide-induced *TP53*-mutant immortalised HUF clones. A confirmed *TP53*-WT was included on each gel to represent a *TP53*-WT response. Representative images of the Western blots are shown. Gapdh and β-actin were used as loading controls. **b** Distribution of glycidamide-induced *TP53* mutations amongst human tumours. Only tumours with more than 1% mutations are shown. NOS, not otherwise specified. **c** *TP53* patterns found in breast, ovarian, lung, colorectal, all tumours, and glycidamide-treated immortalised HUFs. Germline mutations for breast and ovarian cancer were excluded. Reference for human tumour data is the IARC TP53 mutation database (R20, July 2019). Studies recommended being excluded by IARC were not considered

Expression and induction of p53 and its pathway proteins p21 and Mdm2 were assessed in immortal *TP53*-mutant HUFs treated with and without 10 µM Nutlin-3a for 24 h. These results are shown in Fig. 2a and summarised in Supporting Table S2. Lysates of a *TP53*-WT culture treated with Nutlin-3a were included to demonstrate a typical response towards Nutlin-3a treatment. In *TP53*-WT cells, p53, p21, and Mdm2 were induced after treatment with 10 µM Nutlin-3a, with p53 levels being significantly lower than in *TP53 mutants*. As previously shown by Kucab et al. (2017), typical *TP53* mutants express p53 either not at all or very strongly, while p21 and Mdm2 are generally neither expressed nor induced. Most glycidamide-induced *TP53* mutants showed a strong constitutive expression of p53, but no induction after Nutlin-3a treatment (cultures GA-5, -13, -29, -39, -40, -77, -82, -91, -134, -162, -164, -194, -197). Interestingly, GA-194 expressed p53, but the size was decreased to ~ 38 kDa instead of 49 kDa. Three of the remaining mutants did not express any p53 protein (GA-23, -44, -48), which agrees with their p53 functionality (i.e., splice and non-functional). Cultures GA-52, -116, -118, and -196 all expressed p53 weakly, while in culture GA-126, Nutlin-3a treatment induced the expression of p53. In most *TP53*-mutant cultures, p21 and Mdm2 were neither expressed nor induced after Nutlin-3a treatment (GA-5, -23, -29, -39, -40, -44, -48, -52, -77, -82, -91, -118, -134, -162, -194, -197), meaning that those cultures showed a typical *TP53*-mutant response towards Nutlin-3a. In mutants GA-13, -116, -126, -164, -196, the expression of both proteins increased after treatment with Nutlin-3a, which is a typical response observed in *TP53*-WT cultures, but has been observed in some *TP53*-mutant HUFs previously (Kucab et al. 2017). As cultures GA-13, -164, and -196, all harboured *TP53* mutations within exons 4–9 and a *TP53*-WT response was previously reported in *TP53* mutants (Kucab et al. 2017), no other steps were taken for those cultures. However, cultures GA-116 and -126 were examined further as they harboured a mutation outside of exons 4–9 to confirm that they are indeed *TP53* mutants. As cell viability of the Nutlin-3a-treated cultures GA-116 N and -126 N was not decreased after Nutlin-3a treatment, lysates were prepared from those cultures after exposure to Nutlin-3a for 24 h and included for Western blot analysis. The response of GA-116 N and -126 N reflects the response of a typical *TP53*-mutant culture (Fig. S3k). In addition, the response of the remaining cultures that harboured mutations in *Trp53* but not *TP53* (GA-52, -134, and -194) all reflected that of a typical *TP53*-mutant culture. Culture GA-194 expressed p53 strongly as a ~ 38 kDa protein, which agrees with the classification as complex deletion in exon 7 leading to a protein with decreased size. Interestingly, the expression of the loading control Gapdh was affected in cultures GA-39, -40, -52, -134, -162, which was also observed previously in other immortalised HUFs (Kucab et al. 2017). To ensure that protein normalisation was performed correctly, gels were re-probed with a second loading control, namely β-actin, whose expression was uniform in all cultures.

#### Comparison of *TP53* mutations induced by glycidamide with *TP53* mutations found in human tumours

The mutations found after glycidamide treatment in exons 4–9 of the human *TP53* gene in immortalised HUFs were compared with *TP53* mutations found in human tumours listed in the IARC TP53 mutation database (R20, July 2019). All glycidamide-induced *TP53* mutations were listed prior to this study in 3–456 human tumour samples (total 1369). Furthermore, all codons and splice sites targeted by glycidamide were found to be mutated in human cancers (Supporting Table S3). Next, the distribution of the glycidamide-induced *TP53* mutations amongst human tumour types was investigated. The most common cancers with the *TP53* mutations characteristic for the glycidamide-induced mutations were ovarian (10%) and breast (10%) cancer followed by ~ 8% each colorectal and lung cancer (Fig. 2b). The predominant mutation type in all cancer (44%) as well as breast (43%), ovarian (41%), and colorectal cancers (62%) is G > A/C > T, while, in lung cancer, it is 29% G > T/C > A transversion. Whereas G > A/C > T transitions can be the consequence of spontaneous deamination of 5-methylcytosine, G > T/C > A transversions are often caused by misreplication of guanine bases covalently bound to bulky adducts (Kucab et al. 2010). This is followed by 13–15% A > G/T > C in breast and ovarian cancers, respectively, 26% G > A/C > T in lung cancer and 8% G > T/T > A transversions in colorectal cancer. In all cancer cases, the second most common mutation type is 15% G > T/C > A transversions. As described above, the predominant mutation type found in *TP53* in immortalised glycidamide-exposed HUFs was A > T/T > A. This is generally a very uncommon mutation type accounting for only 4–6% of *TP53* mutations in breast, ovarian, and all cancer types. However, A > G/T > C mutations accounted for ~ 18% of the mutations in immortalised HUFs and are common in the tumour types discussed (Fig. 2c).

#### Comparison of *TP53* mutations induced by glycidamide with *TP53* mutations found in previous HIMAs

As shown in Supporting Table S4, most *TP53* mutations found after treatment with glycidamide are unique to glycidamide-treated HUFs and have not been identified previously in immortalised HUFs (cultures GA-5, -13, -23, -44, -48, -77, -82, -91, -118, -196). The *TP53* mutation in cultures GA-162 (K132) and GA-29 (C176) was also found in one spontaneously immortalised HUF culture. Cultures GA-39 and -40 had a mutation in C135, which was found in almost every HIMA that has been performed previously. While the *TP53* mutation in GA-164 (C141) was also found in BaP-treated HUFs, the one identified in GA-197 (V272) was found to be mutated in *N*-methyl-*N*-nitro-*N*-nitrosoguanidine (MNNG)- and BaP-7,8-diol-9,10-epoxide (BPDE)-treated HUFs.

### Whole-genome mutations induced by acrylamide and glycidamide in immortalised HUFs

#### Whole-genome mutation burden after treatment with acrylamide and glycidamide in immortalised HUFs

Single-cell clones were prepared from a selected number of immortalised HUF clones: four individual clones treated with 1.1 mM glycidamide (GA-5, -39, -162, -197), four untreated clones that had immortalised spontaneously and served as controls (SP-1, -2, -3, -4), as well as three individual clones each treated with either 1.5 mM (ACR-1, -2, -3) or 3 mM (ACR-4, -5, -6) acrylamide. DNA of each single-cell clone was successfully whole genome sequenced with a total sequencing depth of ~ 20-fold and aligned to the murine reference genome (*Mus musculus* GRCm38 and ensembl 84 transcriptome). DNA from untreated primary HUFs was also subjected to WGS, and mutations were called against the same embryo as used in the HIMA. Mutations which arose after the single-cell cloning were filtered out by removing variants with a VAF ≤ 0.2. The average SBS burden in the spontaneously immortalised group (i.e., controls) was ~ 8700, while an average of ~ 500 indels (ID) were identified. Clones treated with 1.5 mM acrylamide harboured on average less SBS (~ 5600) and ID (~ 400) than controls. This difference was significant for SBS mutations (one-way ANOVA). A slightly higher SBS mutation burden (~ 14,000) compared with the control was observed following treatment with 3 mM acrylamide, while the number of ID mutations was very similar in both treatment groups. Although the average SBS and ID mutation burdens in glycidamide-treated clones were considerably higher with ~ 11,000 and ~ 600, respectively, it was not significantly different to the control, which was most likely due to the high sample variability within each treatment group. The total days for which the respective clone was kept in culture for isolation of HUFs, immortalisation, and single-cell cloning were also recorded to investigate if any variability in total numbers of SBS and ID per treatment group was related to the time each clone spent in culture. More mutations might arise in clones that have been cultured for a longer time due to reactive oxygen species (i.e., oxidative damage to DNA); however, no clear association regarding the SBS and ID mutation burden and the time which a clone spent in culture was found. A summary of the mutation burden and days spent in culture can be found in Table 3.

**Table 3:** Overview of single base substitution (SBS) and indel (ID) mutation burden and mutation pattern in the whole genome of acrylamide- or glycidamide-treated and spontaneously immortalised (i.e., control) HUFs

	Spontaneous				1.5 mM acrylamide			3 mM acrylamide			1.1 mM glycidamide			
Sample	SP-1	SP-2	SP-3	SP-4	ACR-1	ACR-2	ACR-3	ACR-4	ACR-5	ACR-6	GA-5	GA-39	GA-162	GA-197
Days in culture	130	155	137	135	121	114	99	144	151	125	107	121	97	112
Number SBS	9643	9916	6781	8463	6279	6968	3625	19,648	13,215	10,197	12,230	10,764	9928	10,906
Average SBS	8701				5624			14,353			10,957			
Number ID	335	371	558	700	427	568	203	305	286	333	427	784	372	991
Average ID	491				399			308			644			
Mutation type														
G > T/C > A (%)	14				14			9			15			
G > C/C > G (%)	9				10			7			11			
G > A/C > T (%)	6				7			4			8			
A > T/T > A (%)	15				15			14			21			
A > G/T > C (%)	20				20			22			23			
A > C/T > G (%)	30				28			41			15			
ID (%)	6				7			2			6			
Mutations on NTS^a^ (%)	51				51			49			54			
Mutations on TS^a^ (%)	49				49			51			46			

Shown are the average mutation burden per treatment group and for each clone. In addition, the days in culture for each clone are indicated. Mutations were detected by WGS. Mutations were detected by WGS

^a^ The transcribed (TS) and non-transcribed strands (NTS) were classified for mutations on the purine base

#### Mutation pattern induced by acrylamide and glycidamide in the whole genome in immortalised HUFs

**Fig. 3:**
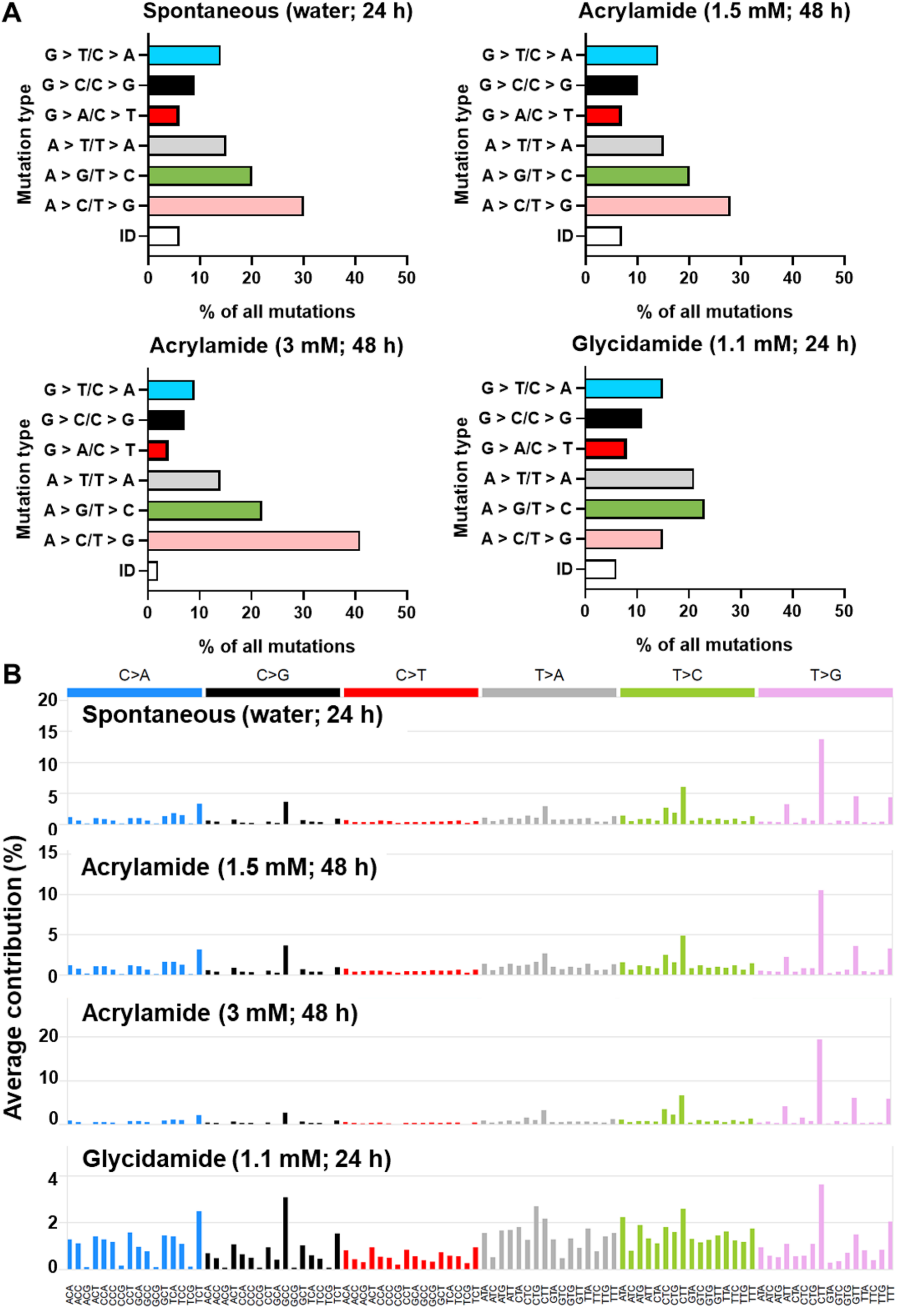
Results of the WGS experiment. **a** Mutation pattern in the whole genome of spontaneously and acrylamide- or glycidamide-treated immortalised HUFs. Shown is the proportion of each mutation type as % of all mutations. **b** Average SBS trinucleotide profiles derived from WGS data from spontaneously and acrylamide- or glycidamide-treated immortalised HUF clones. The trinucleotide sequences are shown in the x-axis, while the y-axis shows the average contribution of the respective mutation to the overall SBS count (%). The six possible pyrimidine substitutions are shown on the top and the peaks indicate the contribution of each mutation to the overall profile

The mutation pattern observed in acrylamide-treated and spontaneously immortalised HUF clones was highly similar (Table 3 and Fig. 3a). The predominant mutation type was ~ 30% A > C/T > G followed by 20% A > G/T > C. Furthermore, 15% A > T/T > A, 14% G > T/C > A, ~ 10% G > C/C > G, and ~ 5% G > A/C > T substitutions each were quantified. In contrast, glycidamide-treated HUF clones showed a distinctly different mutation pattern dominated by 23% A > G/T > C and 21% A > T/T > A mutations followed by 15% A > C/T > G transversions (Fig. 3a). A similar amount of G > A/C > T, G > C/C > G, and G > T/C > A substitutions and IDs as found in spontaneously immortalised and acrylamide-treated clones was also observed in glycidamide-treated clones. Slightly more SBS were on the non-transcribed (54%) than on the transcribed (46%) strand of glycidamide-treated HUFs, while no difference was observed in acrylamide-treated and spontaneously immortalised HUFs (51 vs. 49%). When examining the distribution of each mutation type on the transcribed and non-transcribed strand (Supporting Fig. S4), no difference was present in acrylamide-treated and spontaneously immortalised clones; all SBS were distributed evenly on both the transcribed and non-transcribed strand. Although A > C/T > G transversions were slightly biased towards the non-transcribed strand in all treatment groups, differences were not statistically significant. In glycidamide-treated clones, a significant strand bias was found for A > T/T > A transversions, which is partially consistent with another study with glycidamide performed in the exome of HUFs that found strand-biased A > T/T > A and A > G/T > C mutations (Zhivagui et al. 2019).

#### SBS trinucleotide profile in immortalised HUFs after treatment with acrylamide and glycidamide

Figure 3b shows the trinucleotide profile average for each treatment group; individual profiles for each clone can be found in the Supporting Data (Fig. S5). The profile in spontaneously immortalised and acrylamide-treated HUFs was found to be highly similar and characterised by A > C/T > G substitutions at the sequence context 5′-NTT-3′ and G > C/C > G transversions at 5′-GCC-3′. In contrast, the profile induced by glycidamide was defined by more A > G/T > C and A > T/T > A in various sequence contexts, while also showing the distinctive peaks at A > C/T > G and G > C/C > G also seen in the spontaneously immortalised and acrylamide-treated clones.

#### Mutational signature analysis in immortalised HUFs after treatment with acrylamide and glycidamide

**Fig. 4:**
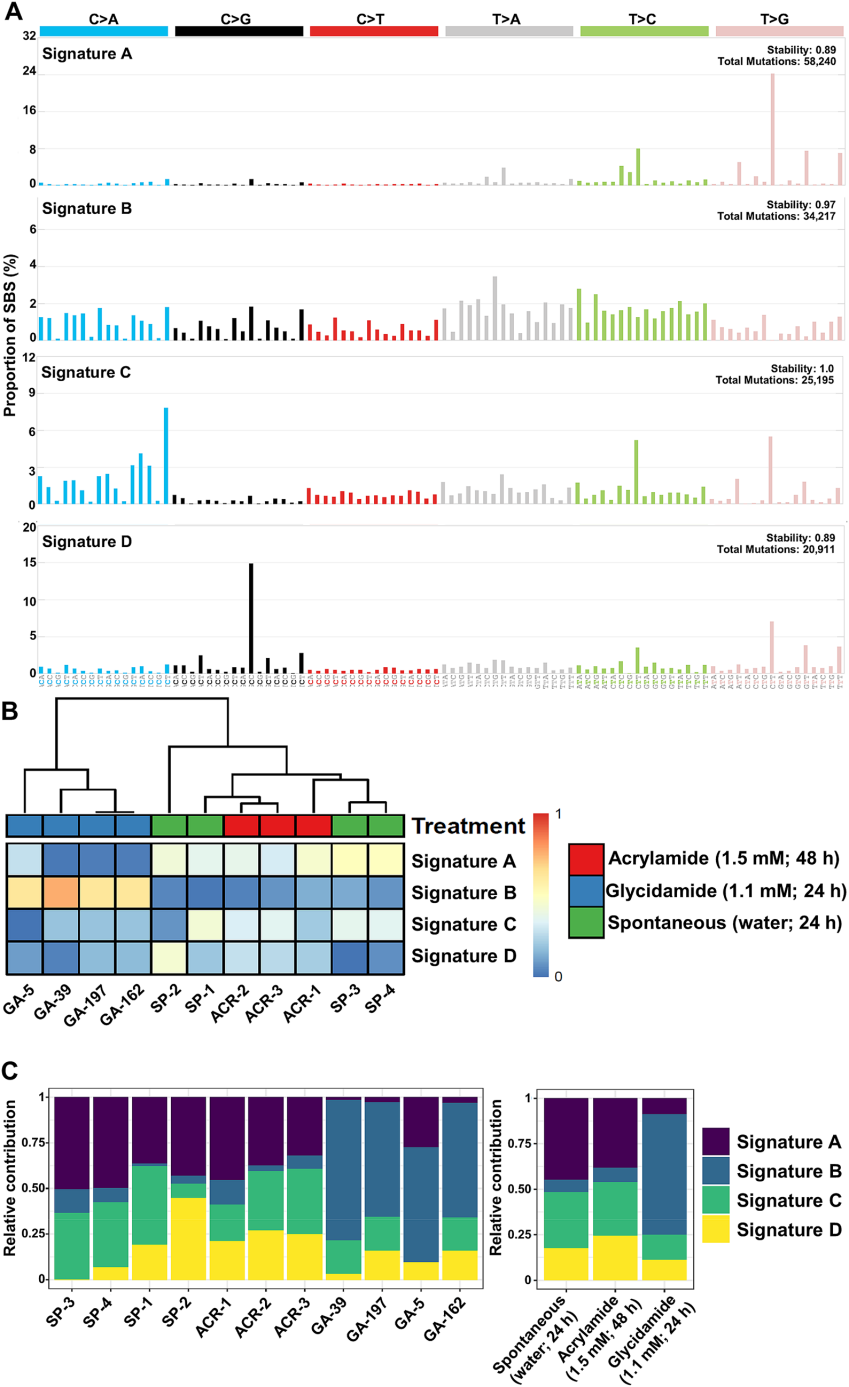
Mutational signature extraction. **a** Extraction of four mutational signatures using SigProfiler. The trinucleotide sequences are shown in the x-axis, while the y-axis shows the proportion of the respective mutation to the overall SBS count (%). The six possible pyrimidine substitution are shown on the top and the peaks indicate the contribution of each mutation to the overall signature. **b** Hierarchical clustering of each signature and c relative contribution of signatures to each sample and treatment group

In the immortalised HUF clones subjected to WGS, signatures of four mutational processes were selected as the optimal number of *N*. The extracted signatures were normalised to the human whole-genome context and compared to the 49 SBS signatures as found in the COSMIC database. The extracted mutational signatures A–D are shown in Fig. 4a. Signatures A, C, and D were present in all samples and more prominent in spontaneously immortalised and acrylamide-treated HUF clones, while signature B was enriched and distinct in glycidamide-treated HUF clones. With regards to mutational signatures, all glycidamide-treated clones clustered together in the heatmap, while acrylamide-treated clones clustered with the spontaneously immortalised HUF clones (Fig. 4b). Interestingly, Signature B was also present in all spontaneously and acrylamide-treated immortalised HUFs, but its overall contribution was very low compared to HUFs immortalised after glycidamide treatment (Fig. 4c).

Signatures A, C, and D are most likely related to culture conditions. Signature A is characterised by A > C/T > G substitutions in the sequence context 5′-NTT-3′ and this has been observed previously in immortalised HUF clones (Nik-Zainal et al. 2015; Zhivagui et al. 2019). This signature has a cosine similarity of 0.93 with COSMIC SBS17b. COSMIC SBS17 has been previously split into COSMIC SBS17a and b and has been observed in human cancer, particularly in oesophageal and gastric adenocarcinoma (Alexandrov et al. 2020; Tomkova et al. 2018), but also in other immortalised HUF clones (Nik-Zainal et al. 2015; Zhivagui et al. 2019), in 5-fluorouracil-treated human intestinal organoids (Christensen et al. 2019), and in aflatoxin B1-induced murine liver tumours (Huang et al. 2017). SBS17 has been linked to oxidative stress, which can result from cell culture conditions or gastric reflux in human gastric and oesophageal tumours. Signature C is characterised by G > T/C > A, especially at 5′-TCT-3′, A > G/T > C, predominantly at 5′-CTT-3′, and A > C/T > G at 5′-CTT-3′. Signature D is defined by G > C/C > G transversions and shows a preference for sequence context 5′-GCC-3′. A similar signature has been observed previously by WES in immortalised HUF clones (Olivier et al. 2014; Zhivagui et al. 2019). G > C/C > G transversions are also the predominant mutation type in *TP53* of HUFs spontaneously immortalised (Whibley et al. 2010) and have been related to culture conditions (Olivier et al. 2014). Signatures C and D show no cosine similarity higher than 0.80 to any of the 49 SBS COSMIC signatures.

**Fig. 5:**
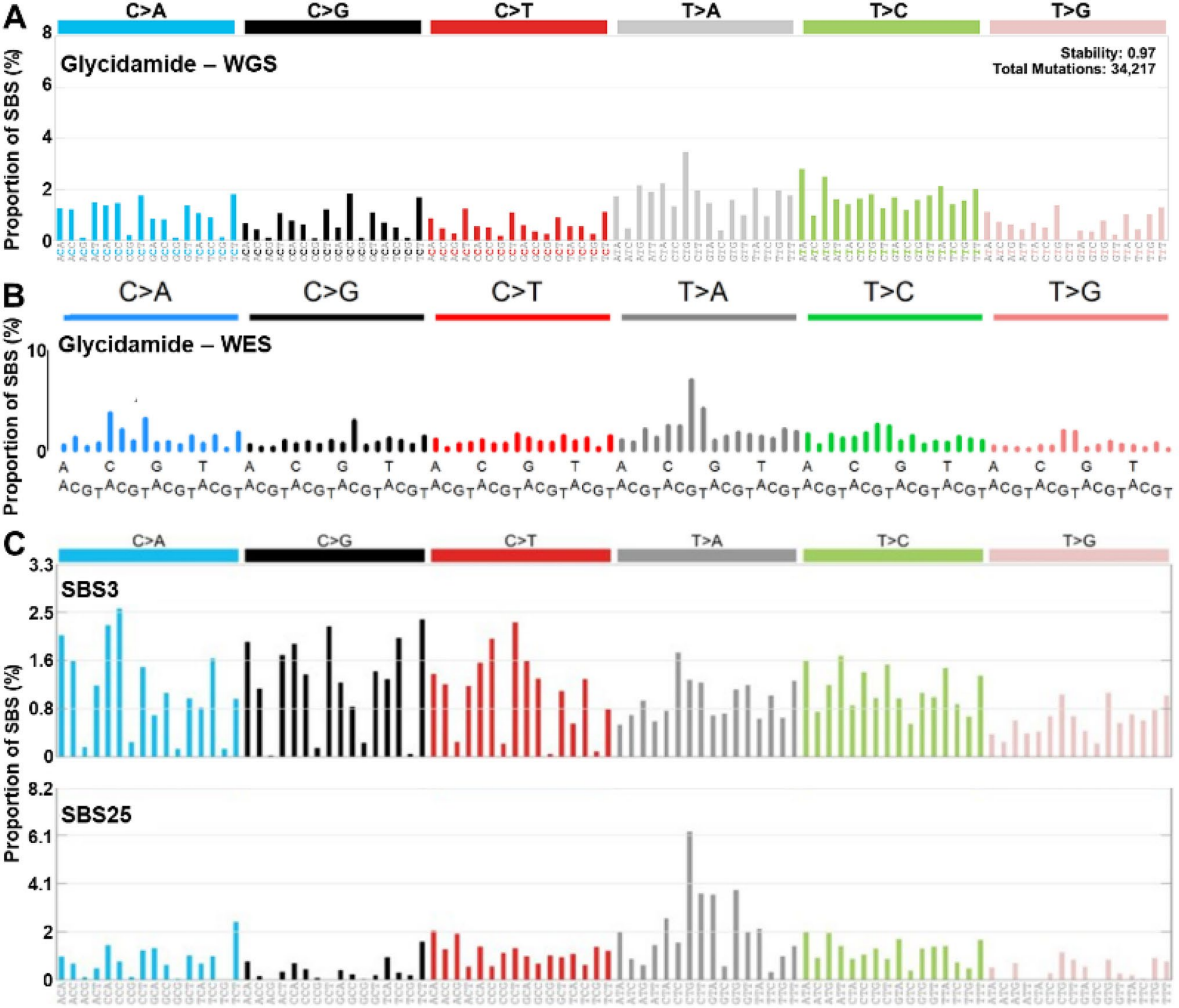
Comparison of WGS mutational signatures with the literature and COSMIC database. **a** WGS mutational signature extracted from glycidamide-treated HUF clones (present study). **b** WES mutational signature extracted from glycidamide-treated HUF adapted clones from Zhivagui et al. 2019. **c** COSMIC SBS3 that has been proposed to be associated with defective homologous recombination-based DNA damage repair, which manifests predominantly as small indels and genome rearrangements due to abnormal double-strand break repair but also in the form of this base substitution signature. Adapted from the COSMIC database (V3, May 2019). COSMIC SBS25 with unknown aetiology. However, some Hodgkin’s cell line samples in which the signature has been found were from patients exposed to chemotherapy and it is possible that SBS25 is due to chemotherapy treatment. Adapted from the COSMIC database (V3, May 2019)

The glycidamide-related signature B is characterised by a diverse mutational pattern and sequence context with more SBS at A:T than at G:C base pairs, which is consistent with the mutation pattern observed in the *TP53* gene. The most prominent mutation types are A > T/T > A at the sequence context 5′-CTG-3′ and A > G/T > C at various sequence contexts, which were also the predominant types in the *TP53* gene in glycidamide-treated HUFs. Next, the extracted WGS signature was normalised to the human whole-genome context and compared to the 49 COSMIC SBS signatures. Signature B was similar to COSMIC SBS3 and 25 with a calculated cosine similarity of 0.82 and 0.85, respectively (Fig. 5c). SBS3 is observed in breast, pancreatic, and ovarian cancers, and was related to somatic and germline *BRCA1/2* mutations as well as defective homologous recombination (HR) base repair (Alexandrov et al. 2020). In contrast, the aetiology of COSMIC SBS25 remains unknown, but it has only been found in Hodgkin lymphoma cell lines of patients exposed to chemotherapy. Thus, it is likely that SBS25 is caused by chemotherapy treatment. The normalised signature B was compared to a glycidamide-related signature previously extracted by WES in immortalised HUF clones (Zhivagui et al. 2019) and a cosine similarity of 0.91 was calculated indicating a strong similarity to the signature identified by the WGS analysis (Fig. 5b). The WES signature was observed in one-third of 1600 cancer types studied and was most commonly found in lung, liver, and kidney cancer (Zhivagui et al. 2019). However, similarities between the experimental mutational signature of glycidamide and mutational signature extracted from human tumours provide the evidence of an association and not of causation.

Kucab et al. (2019) generated a compendium of mutational signatures of environmental agents by exposing human pluripotent stem cells to 79 known or suspected environmental carcinogens. After WGS, 41 of them yielded characteristic substitution mutational signatures in these cells, with some being similar to those found in human tumours. Although this is speculative and the number of mutational signatures generated from other mutagens is at present fairly limited, none of them is similar to that of glycidamide found in exposed HUFs (cosine similarities 0.79 or less; see Table S5).

### Further analyses of acrylamide-treated immortalised HUF clones

While the higher number of SBS in HUFs immortalised after treatment with 3 mM acrylamide (Table 3) indicated that acrylamide-specific SBS mutations were induced, the mutation pattern was very similar to that observed in spontaneously immortalised HUFs and HUFs immortalised after treatment with 1.5 mM acrylamide (Table 3 and Fig. 3a). When taking the flanking bases into account, no difference between the two treatment groups (i.e., 3 mM acrylamide and spontaneously immortalised) was observed and the SBS trinucleotide profiles were characterised by A > C/T > G at the 5′-NTT-3′ sequence context and G > C/C > G mutations as observed in immortalised HUFs treated with 1.5 mM acrylamide (Fig. 3b). As no difference between the average trinucleotide profiles of the treatment groups was observed, mutational signature analysis was not performed for immortalised HUFs treated with 3 mM acrylamide.

## Discussion

Understanding acrylamide’s role in human tumour development is crucial as humans are widely exposed to acrylamide and it remains unclear if acrylamide is carcinogenic to humans or not. Finding specific mutation patterns can help to relate acrylamide exposure to certain human tumour types and better understand underlying mutagenic processes. Thus, the objective of this study was to assess the mutagenicity of acrylamide and its reactive metabolite glycidamide in primary HUFs. We hypothesised that acrylamide and glycidamide leave characteristic mutation patterns in the *TP53* gene and across the whole genome of primary HUFs that could relate to human tumour data from the IARC TP53 mutation and COSMIC databases.

In glycidamide-treated HUFs, the overall mutation frequency in *TP53* was 9% and increased numbers of SBS and ID mutations relative to controls (untreated) were observed in the whole genome. The mutation pattern was characterised by A > T/T > A and A > G/T > C substitutions in both *TP53* (Supporting Fig. S6e) and at whole-genome level (Supporting Fig. S6f). The previous studies have shown that the mutation pattern induced by glycidamide in a variety of experimental systems is complex. Besaratinia and Pfeifer (2004) named G > T/C > A as the hallmark mutation, accounting for 35% of the total number of mutations in the *cII* transgene of glycidamide-exposed Big Blue^®^ MEFs (Supporting Fig. S6a). It is surprising that such a different mutation pattern was found, given that the present study also used MEFs, although isolated from a different mouse strain. The observed differences could be related to the fact that *cII* is a gene of the lambda phage randomly introduced into the mouse genome whereas *TP53* sequenced in this project is an actively transcribed human gene with an important function in carcinogenesis. In contrast to the *cII* gene, the *Hupki* allele retains the transcriptional regulation of *Trp53* in HUFs, which could lead to differences in the formation and removal of DNA adducts in MEFs after carcinogen (e.g., glycidamide) treatment. While some other studies examining the mutation pattern in the *cII* gene of Big Blue^®^ mice and rats identified G > T/C > A as the main mutation type induced by acrylamide, others found A > T/T > A transversions to be more common (see Supporting Fig. S6) (Manjanatha et al. 2006, 2015; Mei et al. 2010). Overall, the previous studies indicate that glycidamide induces a diverse spectrum of mutations dependent on the tissue examined and the experimental system used.

In general, the predominant mutation types identified in *TP53* in glycidamide-exposed HUFs are uncommon in*TP53* of human tumours, but were nevertheless found many times in the IARC TP53 mutation database. For instance, A > T/T > A transversions only account for 6%, A > G/T > C for 11% and G > C/C > G for 7% in all human tumour types. G > C/C > G transversions are the most common (~ 50%) mutation type harboured in spontaneously immortalised HUFs (Whibley et al. 2010), while at the whole genome level, the proportion of G > C/C > G transversion is very similar in glycidamide-treated and spontaneously immortalised HUFs (present study). Moreover, all mutations of this type were based at sequence contexts of the *TP53* gene (i.e., C135, C141) that were discovered many times prior to this study in immortalised HUFs after exposure to other carcinogens. Thus, this mutation type and gene location seems unspecific to glycidamide exposure and possibly occurred spontaneously. In contrast, all observed A > T/ T > A and A > G/T > C mutations, including their sequence context, were not found in immortalised HUFs prior to this study. The fact that these mutation types are rare in both spontaneously immortalised HUFs and immortalised HUFs treated with other agents (Hölzl-Armstrong et al. 2019) but increased in glycidamide-treated HUFs (present study), suggests that they are caused directly by glycidamide exposure. It is of interest to note that the cancer types closely reflecting glycidamide-induced *TP53* mutations were human breast, ovary, lung, and colorectal tumours. While human breast and ovarian cancers have been reported to be associated with dietary acrylamide exposure (Hogervorst et al. 2007; Olesen et al. 2008), colorectal, and lung cancer do agree with routes of acrylamide exposures via diet and tobacco smoking, respectively. The higher than average percentage of A > G/T > C substitutions in breast and ovarian cancer could be related to acrylamide exposure as this mutation type is linked to the N3-GA-Ade DNA adduct induced by glycidamide (Gamboa da Costa et al. 2003). Moreover, the extracted WGS mutational signature for glycidamide showed a cosine similarity of 0.82 with COSMIC SBS3, a signature that is predominantly found in breast and ovary tumours, which further strengthen the hypothesis that glycidamide may impact the formation of these tumour types. However, SBS3 arises most likely due to defects in HR repair and is strongly associated with *BRCA1/2* mutations (Alexandrov et al. 2020). Nevertheless, it cannot be ruled out that acrylamide-exposure plays at least partly a role in the aetiology of SBS3. In the future, the WGS mutational signature induced by glycidamide could be compared with WGS data of human tumours such as those found in the pan-cancer analysis of whole genomes (PCAWG). Furthermore, newly extracted WGS mutational signatures found in human tumours should be included for comparison in future studies. This may help to clarify the currently inconsistent evidence for an association between acrylamide exposure and human cancer development.

Glycidamide-induced *TP53* mutations were found in lung cancer, which is important as acrylamide is present in mainstream tobacco smoke (Mojska et al. 2016; Smith et al. 2000). COSMIC SBS4 is caused by tobacco smoking and characterised by G > T/C > A and A > T/T > A transversions. A visual comparison of SBS4 and the glycidamide signature indicated similarities, especially for the A > T/T > A distribution; however, the calculated cosine similarity of 0.55 is low. It could be speculated that glycidamide contributes to the distribution of A > T/T > A mutations found in SBS4, which could arise in human lungs due to acrylamide exposure from cigarette smoke. The G > T/C > A component of SBS4 was replicated in the whole genome of immortalised HUFs and human iPSCs after BaP exposure (Kucab et al. 2019; Nik-Zainal et al. 2015), while the A > T/T > A component was similar to that induced by dibenzo[*a,l*]pyrene, a polycyclic aromatic hydrocarbon that forms adenine adducts (Kucab et al. 2019). As cigarette smoke is a complex mixture of many chemicals, the composition of SBS4 is most likely due to several mutagens, of which acrylamide could be contributing to the A > T/T > A component of SBS4. Notably, a study examining the exomes of immortalised glycidamide-treated HUFs found a high similarity (cosine similarity = 0.94) of the adenine components of COSMIC SBS4 and the mutational signature of glycidamide extracted by WES (Zhivagui et al. 2019). As the glycidamide signature observed by Zhivagui et al. (2019) by WES and the glycidamide signature extracted in the present study by WGS showed 0.91 cosine similarity, the adenine distribution is probably similar in both. Thus, the evidence suggests that acrylamide is likely to contribute to lung cancer development in smokers.

*TP53* mutations observed in human cancers also often affect p53 function, which can result either in the loss or a gain of function and subsequently can interfere with the tumour suppressor abilities of p53. Most *TP53* mutations identified in immortalised HUFs in the present study led to non-functional p53, indicating that they are driver-mutations rather than functional mutants that are passenger mutations (Hainaut and Pfeifer 2016). Furthermore, while only one *TP53* mutation was found at a hotspot codon (R245), the most commonly mutated codon outside the DBD of *TP53* (Y220) (Hainaut and Pfeifer 2016) was mutated twice in this study. Altogether, the pattern and distribution of *TP53* mutations generated in this study indicate that they are specific to glycidamide exposure and not random, which is an important consideration for cancer aetiology.

The mutation patterns induced by glycidamide in *TP53* and the whole genome are both consistent with the formed glycidamide-DNA adducts. N3-GA-Ade and N7-GA-Gua are depurinating adducts leading to A > T/T > A and G > T/C > A transversions, respectively. The high level of A > T/T > A transversions is explicable by the fact that adducts at the N3 position of adenine usually undergo spontaneous depurination at faster rates than those at the N7 position of guanine. The small amount of G > T/C > A mutations can be explained by the fact that N7-guanine adducts show generally lower mutagenicity (Koskinen and Plna 2000). Although the amount of N3-GA-Ade adducts were not quantified in HUFs in this study, it can be assumed that they were formed: the formation of N7-GA-Gua has been shown to predict the formation of N3-GA-Ade (Gamboa da Costa et al. 2003) and it has been suggested to use N7-guanine adducts as a surrogate biomarker for the other adducts due to their higher abundance and easier quantification (Koskinen and Plna 2000). The high levels of A > G/T > C mutations could be explained by the high mutagenic potential of adducts at the N1 position of deoxyadenosine, since they directly interfere in base pairing regions of the DNA (Koskinen and Plna 2000). The detected concentration-dependent formation of the N7-GA-Gua in glycidamide-treated primary HUFs together with the absence of DNA adducts after acrylamide treatment agrees with the other in vitro studies using mammalian cells (Martins et al. 2007; Mei et al. 2008b; Zhivagui et al. 2019). In another study using primary HUFs, the N7-GA-Gua adduct was only formed after glycidamide and acrylamide + S9 but not acrylamide-S9 treatment (Zhivagui et al. 2019). In contrast to the present study, Zhivagui et al. (2019) detected N7-GA-Gua in glycidamide-treated HUFs at levels three magnitudes higher. However, while the LC–MS/MS method used for the detection and quantification of DNA adducts was based on the same principle as ours, it included an isotope-labelled adduct standard, which provides more accuracy.

While it was possible to extract a glycidamide-related mutational signature by WGS, acrylamide-treated clones showed the same spectrum of mutations as spontaneously immortalised HUFs and only harboured culture-related signatures. Although HUFs treated with 3 mM acrylamide showed an elevated, but not statistically significant, SBS mutation count in comparison to the controls (untreated), still no WGS mutational signature related to acrylamide exposure could be extracted. This is consistent with a previous study that performed WES of acrylamide- and glycidamide-treated HUFs, where a glycidamide-specific signature, but not an acrylamide-specific signature was reported (Zhivagui et al. 2019). Moreover, the observed mutational signatures for glycidamide identified by WGS and WES in both studies showed a cosine similarity of 0.91, indicating that both approaches lead to similar results. The absence of a WGS mutational signature after treatment with 3 mM acrylamide is somewhat surprising as strong induction of DDR proteins along with a significant increase of *lacZ* mutants was observed, indicating that some DNA damage had occurred despite the lack of detectable DNA adduct formation (i.e., N7-GA-Gua). However, the induced DDR can be due to alternative genotoxic mechanisms other than N7-GA-Gua formation as shown previously in human iPSCs in which induction of DDR proteins was observed after acrylamide + S9 treatment, but no WGS mutational signature could be extracted (Kucab et al. 2019). The same study also subjected glycidamide-exposed iPSCs to WGS, but no glycidamide-specific mutational signature could be extracted under the experimental conditions used.

The greater sensitivity of HUFs towards glycidamide than acrylamide is consistent with studies in the other in vitro systems (Baum et al. 2005; Koyama et al. 2006; Mei et al. 2008b). Whereas glycidamide had a concentration-dependent impact on all cellular endpoints assessed prior to the HIMA acrylamide-induced cytotoxicity in a concentration-dependent manner, but no DNA adducts were detected. Statistically increased *lacZ* mutant frequency only occurred after treatment at the highest acrylamide concentration tested (3 mM), and this concentration was also the only one to induce strong γ-H2ax protein expression as well as high cytotoxicity, which could be due to increased cell death. The lack of mutagenicity after acrylamide treatment is not surprising as acrylamide has shown low reactivity towards DNA in vitro (Friedman 2003) and treatment with acrylamide in vivo has led to the formation of only glycidamide-DNA adducts (Segerback et al. 1995). In the 1990s, glycidamide was discovered as a metabolite of acrylamide (Sumner et al. 1992) and was later shown to be mutagenic in vitro (Paulsson et al. 2003). The enzyme catalysing the biotransformation of acrylamide to glycidamide is CYP2E1 (Sumner et al. 1999) and its important role in genotoxicity has been demonstrated in *Cyp2e1* knock out mice where acrylamide is not mutagenic (Ghanayem et al. 2005). While it has been shown previously that HUFs express some CYP enzymes (e.g., Cyp1a2 and 1b1) (Kucab et al. 2012; Liu et al. 2004), the inducibility of *Cyp2e1* in primary HUFs has not been investigated. Cyp2e1 is predominantly expressed in the mouse liver (Renaud et al. 2011), which is why it was not expected to be expressed in primary HUFs at sufficient levels that would allow effective bioactivation of acrylamide. Here, a significant induction of *Cyp2e1* was only observed after treatment with the highest acrylamide concentration tested (3 mM), which is in line with the observed *lacZ* mutagenicity and DDR induction at only that concentration. Because N7-GA-Gua adduct formation was not detected after exposure to 3 mM acrylamide, collectively, these results suggest that the induction of *lacZ* mutagenicity and DDR proteins could be due to other mechanisms (e.g., induction of reactive oxygen species and oxidative damage to DNA or Michael-type nucleophilic addition reactions) (Blasiak et al. 2004; Friedman 2003; Jiang et al. 2007). It needs to be mentioned that CYP2E1 regulation is complex and xenobiotics can increase CYP2E1 expression by enhancing translational efficiency as well as protein stabilisation (Mei et al. 2008a). Thus, to fully determine the enzymatic capabilities of HUFs, it would be important to determine Cyp2e1 protein levels in addition to gene expression, although this was beyond the scope of the current study.

In conclusion, this study found a distinctive *TP53* mutation pattern alongside a novel WGS mutational signature in HUF clones immortalised after glycidamide exposure. Both the *TP53* mutation pattern and the mutational signature induced by glycidamide were characterised by A > T/T > A and A > G/T > C mutations. *TP53* mutations characteristic of those induced by glycidamide occur in human tumours including breast, ovary, colorectal, and lung. The novel WGS mutational signature related to glycidamide exposure showed similarity to COSMIC SBS3 and 25 and the WES mutational signature recently extracted in glycidamide-treated immortalised HUFs by Zhivagui et al. (2019). In addition, primary HUFs were clearly more sensitive towards glycidamide than acrylamide treatment, which is most likely due to a lack of efficient metabolic activation of acrylamide to glycidamide. Overall, this study has helped to further understand the mutagenic mechanisms of acrylamide and its metabolite glycidamide. Comparing the WGS mutational signature induced by glycidamide in HUFs to mutational signatures extracted from human tumours in the future linked to environmental exposure to acrylamide may help to clarify its role in human carcinogenesis.

## Electronic supplementary material

Below is the link to the electronic supplementary material.

Supplementary file1 (PDF 1592 kb)

## Abbreviations

3-NBA: 3-Nitrobenzanthrone
AA I: Aristolochic acid I
ANOVA: Analysis of variance
AUC: Area under the curve
BaP: Benzo[*a*]pyrene
BPDE: Benzo[*a*]pyrene-7,8-diol-9,10-epoxide
COSMIC: Catalogue Of Somatic Mutations In Cancer
CYP/Cyp: Cytochrome P450 (human/mouse)
DDR: DNA damage response
DMSO: Dimethyl sulfoxide
GAPDH/Gapdh: Glyceraldehyde 3-phosphate dehydrogenase
HIMA: Hupki mouse embryo fibroblast immortalisation assay
HUF: Hupki mouse embryo fibroblast
Hupki: Human *TP53* knock-in
IARC: International Agency for Research on Cancer
ID/Indel: Insertions and deletions
iPSC: Induced pluripotent stem cells
IPTG: Isopropyl-β-D-thiogalactopyranoside
LLOQ: Lower limit of quantification
MDM2/Mdm2: Murine double minute 2
MEF: Mouse embryo fibroblast
MNNG: *N*-Methyl-*N*-nitro-*N*-nitrosoguanidine
MUT: Mutant
N1-GA-dA: N1-(2-carboxy-2-hydroxyethyl)-2′-deoxyadenosine
N3-GA-Ade: N3-(2-carbamoyl-2-hydroxyethyl)adenine
N7-GA-Gua: N7-(2-carbamoyl-2-hydroxyethyl)guanine
NGS: Next-generation sequencing
NMF: Non-negative matrix factorisation
PBS: Phosphate-buffered saline
PCAWG: Pan-cancer analysis of whole genomes
PCR: Polymerase chain reaction
P-gal: Phenyl-β-D galactosidase
SBS: Single base substitution
SD: Standard deviation
SSB: Single-strand break
*TP53/Trp53*: Tumour protein gene 53 (human/mouse)
UPLC-ESI–MS/MS: Ultra-performance liquid chromatography coupled with electrospray ionisation tandem mass spectrometry
WES: Whole-exome sequencing
WGS: Whole-genome sequencing
WT: Wild-type
X-gal: 5-Bromo-4-chloro-3-indolyl-D-galactopyranoside
