# Supplementary material for: Mutagenicity of acrylamide and glycidamide in human TP53 knock-in (Hupki) mouse embryo fibroblasts
Source: Arch Toxicol. 2020 Sep 4;94(12):4173–96. doi: 10.1007/s00204-020-02878-0 (PMC7655573; doi:10.1007/s00204-020-02878-0)
Supplement: Supplementary file 1 — Supplementary file1 (PDF 1592 kb) [file 204_2020_2878_MOESM1_ESM.pdf]

# Electronic Supplementary Material, Archives of Toxicology

## Mutagenicity of acrylamide and glycidamide in human *TP53* knock-in Hupki mouse embryo fibroblasts

Lisa Hölzl-Armstrong[1], Jill E. Kucab[1], Sarah Moody[2], Edwin P. Zwart[3], Lucie Loutkotová[4,5], Veronica Duffy[1], Mirjam Luijten[3], Gonçalo Gamboa da Costa[4], Michael R. Stratton[2], David H. Phillips[1,†] and Volker M. Arlt[1,6,†]

[1] Department of Analytical, Environmental and Forensic Sciences, MRC-PHE Centre for Environment and Health, King's College London, London, SE1 9NH, UK

[2] Cancer, Ageing and Somatic Mutation, Wellcome Trust Sanger Institute, Hinxton, CB10 1SA, UK

[3] Center for Health Protection, National Institute for Public Health and the Environment (RIVM), Bilthoven, 3720, The Netherlands

[4] Division of Biochemical Toxicology, National Center for Toxicological Research, U.S. Food and Drug Administration, Jefferson, Arkansas, 72079, USA

[5] Present Address: Covance Inc., Salt Lake City,Utah, 84124, USA

[6] Present Address: Toxicology Department, GAB Consulting GmbH, 69126 Heidelberg, Germany

[†] These authors contributed equally

Corresponding author: Dr. Volker M. Arlt, Present Address: GAB Consulting GmbH, Toxicology Department, 69126 Heidelberg, Germany. E-mail: volker.arlt@kcl.ac.uk

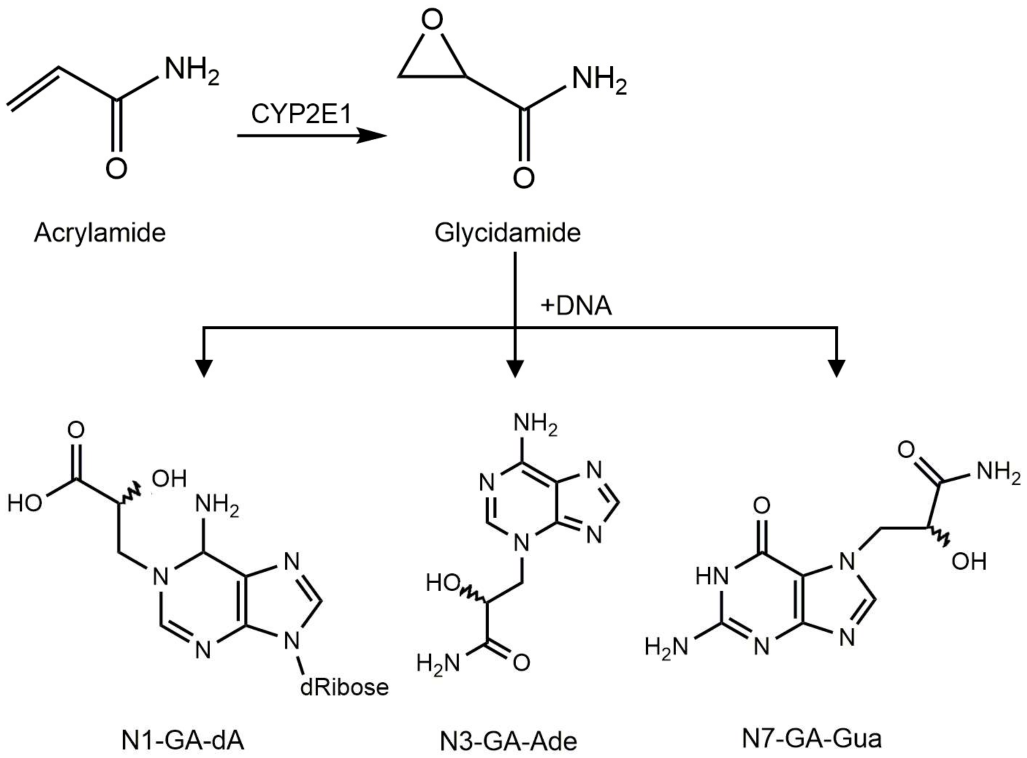


**Fig. S1** Metabolic activation pathway of acrylamide and the three main adducts formed by glycidamide. N7-GA-Gua, N7-(2-carbamoyl-2-hydroxyethyl)guanine; N3-GA-Ade, N3-(2-carbamoyl-2-hydroxyethyl)adenine; N1-GA-dA, N1-(2-carboxy-2-hydroxyethyl)-2'-deoxyadenosine.

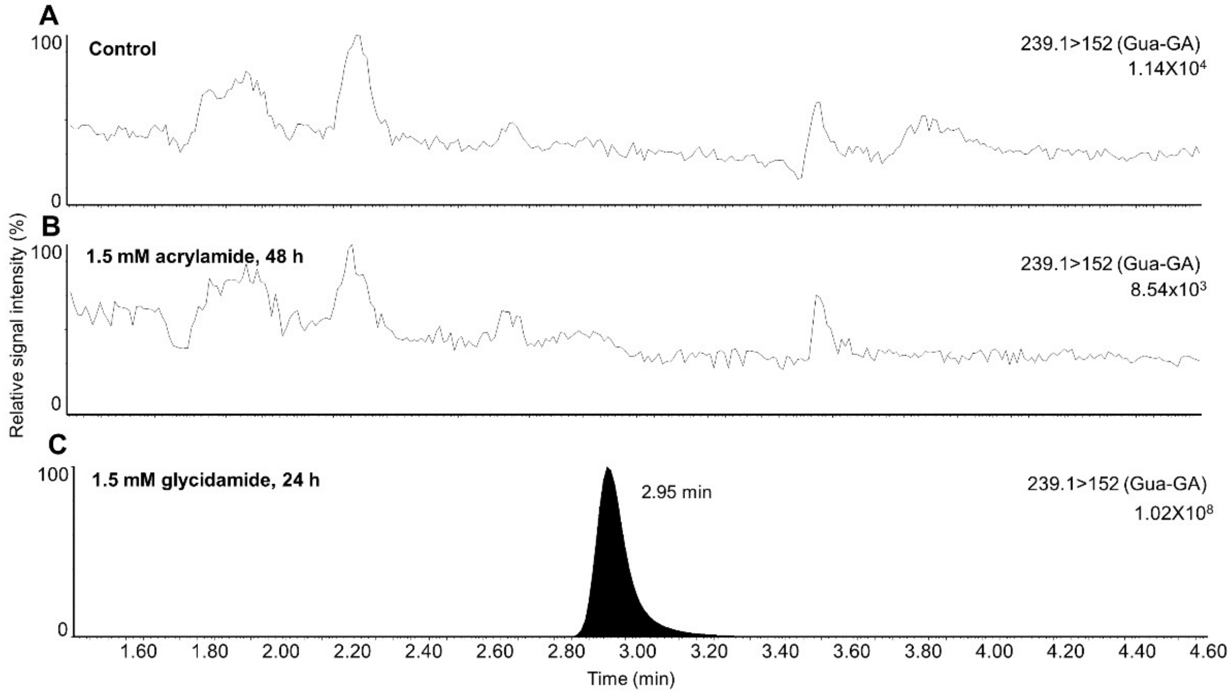


**Fig. S2** Representative chromatograms from the UPLC-ESI-MS/MS analysis. Primary HUFs were treated with (**a**) water, (**b**) 1.5 mM acrylamide for 48 h or (**c**) 1.5 mM glycidamide for 24 h.

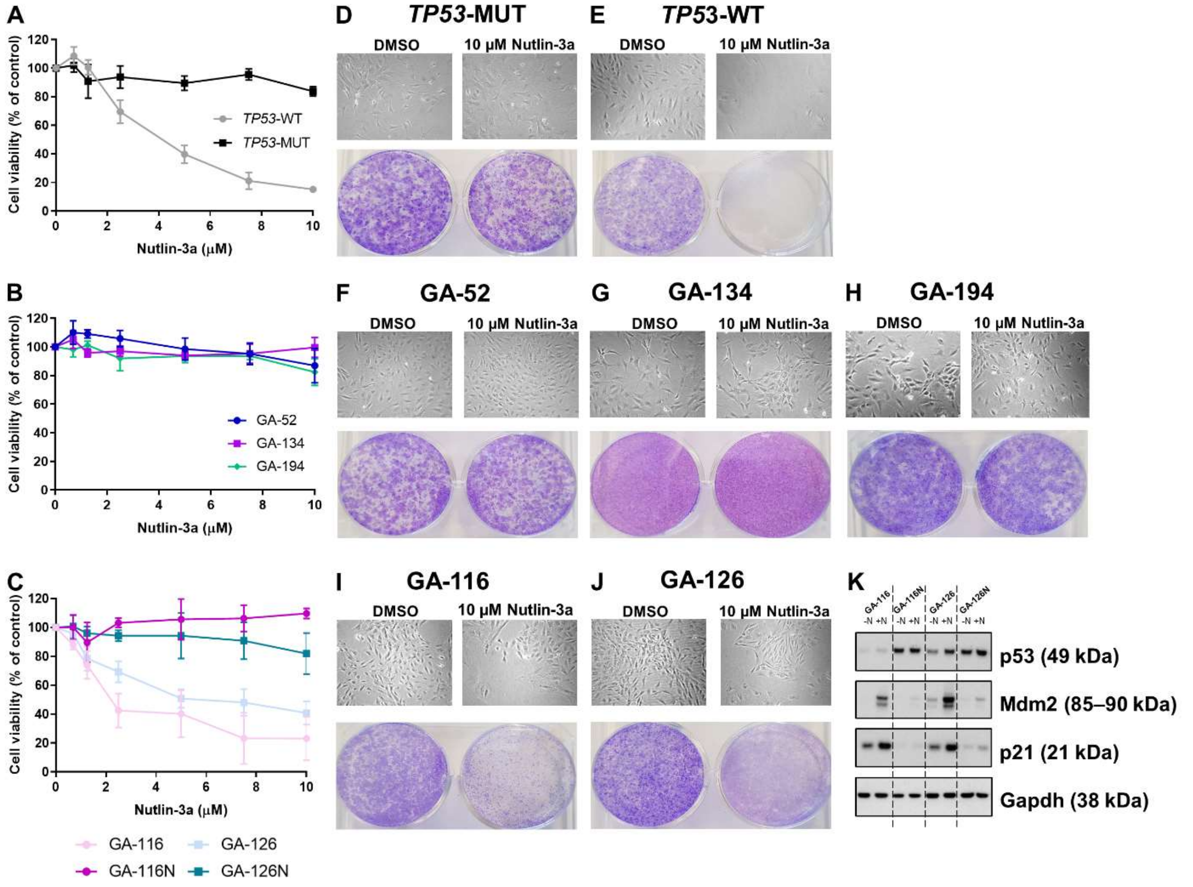


**Fig. S3** Cellular response of cultures with unidentified *TP53* mutations towards Nutlin-3a treatment. **a–c** Cell viability (% control) was assessed after treatment of immortalised HUFs with 0–10 µM Nutlin-3a for five days followed by crystal violet staining Cells treated with 0.1% DMSO served as control. Shown are mean values ± SD. **d–j** Photomicrographs (x100) were taken after treatment with 10 µM Nutlin-3a for five days. Cells were then stained with crystal violet to demonstrate the growth inhibition of Nutlin-3a and photos were taken for documentation Data shown are representative of at least two experiments. **k** Induction of p53 pathway proteins in cultures GA-116N and -126N.

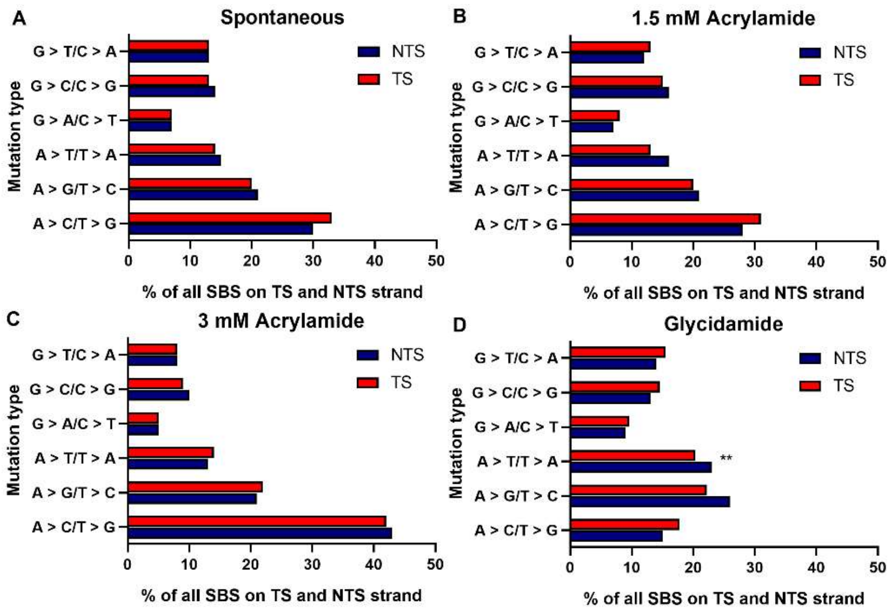


**Fig. S4** Mutation pattern as found on the non-transcribed (NTS) or transcribed (TS) strand in the whole genome of **a** spontaneously and **b–c** acrylamide- or **d** glycidamide-treated immortalised HUFs. Shown is the proportion of each mutation type as % of all mutations on TS and NTS strand. Significance was determined by two-sample *t*-test assuming unequal variances (** $p < 0.01$). TS and NTS are defined by the mutation on the purine base.

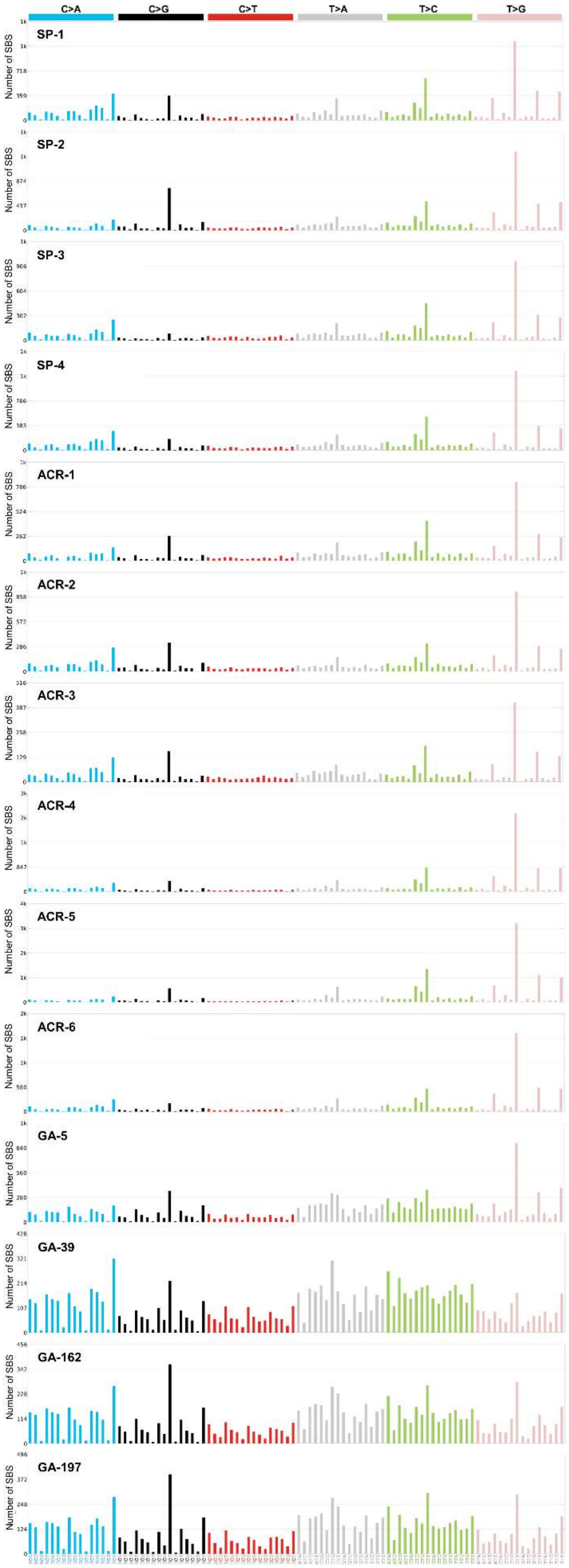
C>A
C>G
C>T
T>A
T>C
T>G
Number of SBS
SP-1
SP-2
SP-3
SP-4
ACR-1
ACR-2
ACR-3
ACR-4
ACR-5
ACR-6
GA-5
GA-39
GA-162
GA-197

**Fig. S5** SBS trinucleotide profiles derived from WGS data from spontaneously and acrylamide- or glycidamide-treated immortalised HUF clones. The trinucleotide sequences are shown on the x-axis, while the y-axis shows the number of the respective SBS. The six possible pyrimidine substitutions are shown at the top and the peaks indicate the number of each SBS.

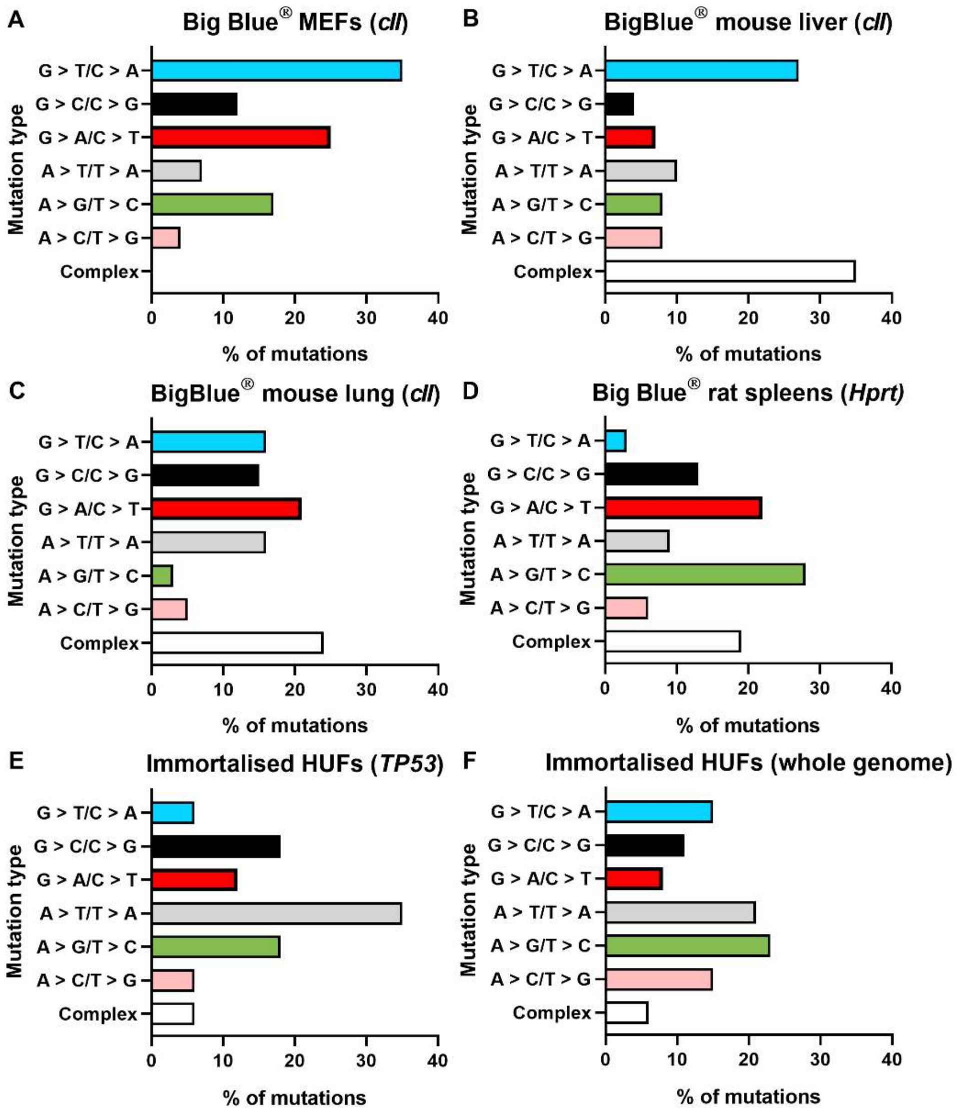


**Fig. S6** Comparison of mutation patterns induced by glycidamide in different experimental systems. Mutation pattern in the *cII* gene of Big Blue® MEFs (**a**, Besaratinia and Pfeifer 2004), the *cII* gene of Big Blue® mouse livers (**b**, Manjanatha et al. 2006), the *cII* gene of Big Blue® mouse lungs (**c**, Manjanatha et al. 2015), the *Hprt* gene of Big Blue® rat spleens (**d**, Mei et al. 2010), the *TP53* gene of glycidamide-treated immortalised HUFs (**e**, present study) and the whole genome of glycidamide-treated immortalised HUFs (**f**, present study).

**Table S1** Cycling parameters for PCR amplification for WGS.

| Step | Cycling parameters | | |
|---|---|---|---|
| | **Temperature (°C)** | **Time (min)** | **Cycles** |
| **1** | 95 | 5 | 1 |
| **2** | 98 | 0.5 | 6 |
| | 65 | 0.5 | |
| | 72 | 1 | |
| **3** | 72 | 10 | 1 |

**Table S2** Summary of the response in glycidamide-induced *TP53*-mutant clones after treatment with 10 µM Nutlin-3a. Protein expression of p53, Mdm2 and p21 in *TP53* mutant clones was assessed by Western blot analysis. The induction of protein is labelled by ↑, no expression by -, no induction ↔. Growth inhibition was assessed following 10 µM Nutlin-3a treatment for 5 days. Activity refers to the activity of the respective mutation found in the yeast promotor assay according to Kato et al. (2003): NF, non-functional; PF, partially functional; NA, not analysed.

| GA- | Coding change | Zygosity | Response towards Nutlin-3a | | | | p53 activity | Gapdh influenced |
|---|---|---|---|---|---|---|---|---|
| | | | Growth inhibition (5 days) | Protein induction (24 h) | | | | |
| | | | | p53 | p21 | Mdm2 | | |
| **5** | Y220C | homo-/hemi- | resistant | ↔ | - | - | NF | no |
| **13** | E286D | hetero- | mixed | ↔ | ↑ | ↑ | NF | no |
| **23** | splice | homo-/hemi- | resistant | - | - | - | NA | no |
| **29** | C176F | hetero- | resistant | ↔ | - | - | PF | no |
| **39** | C135W | homo-/hemi- | resistant | ↔ | - | - | PF | yes |
| **40** | C135W | homo-/hemi- | resistant | ↔ | - | - | PF | yes |
| **44** | R280* | homo-/hemi- | resistant | - | - | - | NA | no |
| **48** | R280* | homo-/hemi- | resistant | - | - | - | NA | no |
| **52** | - | - | resistant | ↔ | - | - | - | yes |
| **77** | H168L | homo-/hemi- | resistant | ↔ | - | - | NF | no |
| **82** | Y205C | hetero- | mixed | ↔ | - | - | NF | no |
| **91** | F270S | homo-/hemi- | resistant | ↔ | - | - | NF | no |
| **116** | - | - | mixed | ↔ | ↑ | ↑ | - | no |
| **118** | E286D | hetero- | resistant | ↔ | - | - | NF | no |
| **126** | - | - | mixed | ↑ | ↑ | ↑ | - | no |
| **134** | - | - | resistant | ↔ | - | - | - | yes |
| **162** | K132T | homo-/hemi- | resistant | ↔ | - | - | NF | yes |
| **164** | C141W | hetero- | mixed | ↔ | ↑ | ↑ | NF | no |

| | | | | | | | | |
|---|---|---|---|---|---|---|---|---|
| **194** | - | - | resistant | ↔ | - | - | - | no |
| **196** | G245S | homo-<br>/hemi- | resistant | ↔ | ↑ | ↑ | NF | no |
| **197** | V272M | homo-<br>/hemi- | resistant | ↔ | - | - | NF | no |

**Table S3** Number of *TP53* mutations in human tumours at *TP53* mutation sites found in immortalised HUFs exposed to glycidamide. Occurrence refers to the number of human tumours harbouring the exact indicated mutation. In addition, the total number of times the respective codon is mutated in human tumours is listed. Total count of mutations is 28,866 (IARC TP53 mutation database, R20, July 2019). Studies recommended to be excluded by IARC are not considered. SA, splice site acceptor.

| GA- | Codon | Mutation | Occurrence | Codon mutation frequency |
|---|---|---|---|---|
| **162** | 132 | A > C/T > G | 6 | 220 |
| **39, 40** | 135 | G > C/C > G | 28 | 268 |
| **164** | 141 | G > C/C > G | 14 | 193 |
| **71** | 168 | A > T/T > A | 9 | 93 |
| **29** | 176 | G > T/C > A | 164 | 396 |
| **76** | 205 | A > G/T > C | 122 | 203 |
| **5** | 220 | A > G/T > C | 402 | 474 |
| **196** | 245 | G > A/C > T | 456 | 868 |
| **85** | 270 | A > G/T > C | 19 | 114 |
| **197** | 272 | G > A/C > T | 114 | 211 |
| **44, 48** | 280 | A > T/T > A | 9 | 306 |
| **13, 118** | 286 | A > T/T > A | 3 | 176 |
| **23** | in. 6 (SA) | A > T/T > A | 23 | 191 |

**Table S4** *TP53* mutations induced by glycidamide compared with *TP53* mutations established in previous HIMAs.

| **Codon/ Intron** | **GA-** | **WT** | **MUT** | **Other HIMAs** | | |
|---|---|---|---|---|---|---|
| | | | | **#** | **Treatment** | **Reference** |
| **in. 6** | 23 | AG | TG | 0 | - | - |
| **132** | 162 | AAG | ACG | 1 | untreated | Whibley et al. 2010 |
| **135** | 39, 40 | TGC | TGG | 1 | *N*-OH-3-ABA | vom Brocke et al. 2009 |
| | | | | 1 | BPDE | Kucab et al. 2015 |
| | | | | 1 | BaP | Reinbold et al. 2008 |
| | | | | 1 | UV | Liu et al. 2004 |
| | | | | 3 | AAI | Nedelko et al. 2009 |
| | | | | 7 | untreated | Whibley et al. 2010 |
| **141** | 164 | TGC | TGG | 1 | BaP | Reinbold et al. 2008 |
| **176** | 29 | TGC | TTC | 1 | untreated | Whibley et al. 2010 |
| **168** | 77 | CAC | CTC | 0 | - | - |
| **205** | 82 | TAT | TGT | 0 | - | - |
| **220** | 5 | TAT | TGT | 0 | - | - |
| **245** | 196 | GGC | AGC | 0 | - | - |
| **270** | 91 | TTT | TCT | 0 | - | - |
| **272** | 197 | GTG | ATG | 1 | MNNG | Nedelko et al. 2009 |
| | | | | 1 | BPDE | Kucab et al. 2015 |
| **280** | 44, 48 | AGA | TGA | 0 | - | - |
| **286** | 13, 118 | GAA | GAT | 0 | - | - |

**Table S5** Comparison of signature B extracted from glycidamide-treated HUFs with signatures found in carcinogen-exposed human iPSC (Kucab et al. 2019). Abbreviations: DBP, dibenzo[*a,l*] pyrene; DBPDE, dibenzo[*a,l*] pyrene diol-epoxide; ENU, *N*-ethyl-*N*-nitrosourea; DMH, 1,2-dimethylhydrazine; 4-ABP, 4-aminobiphenyl; MNU, N-methyl-N-nitrosourea; 1,8-DNP, 1,8-dinitropyrene; DBA, dibenz[*a,h*]anthracene; DBAC dibenz[*a,j*]acridine; MX, 3-chloro-4-(dichloromethyl)-5-hydroxy-2(5H)-furanone; DBADE, dibenz[*a,h*]anthracene diol-epoxide; PhIP, 2-amino-1-methyl-6-phenylimidazo[4,5-*b*]pyridine.

| Compound | Signature B |
|---|---|
| 6-Nitrochrysene (50 µM) + S9 | 0.79 |
| 6-Nitrochrysene (50 µM) | 0.77 |
| 6-Nitrochrysene (0.78 µM) | 0.75 |
| 6-Nitrochrysene (12.5 µM) + S9 | 0.74 |
| AZD7762 (1.625 µM) | 0.72 |
| DBPDE (0.000625 µM) | 0.70 |
| Aristolochic acid II (37.5 µM) | 0.68 |
| ENU (400 µM) | 0.68 |
| DBP (0.0039 µM) | 0.68 |
| Dimethyl sulfate (0.078 mM) | 0.66 |
| DBPDE (0.000156 µM) | 0.65 |
| DBP (0.0313 µM) + S9 | 0.64 |
| 3-NBA (0.1 µM) | 0.62 |
| Propylene oxide (10 mM) | 0.61 |
| Diethyl sulfate (0.938 mM) | 0.60 |
| Ellipticine (0.375 µM) + S9 | 0.59 |
| Aristolochic acid I (1.25 µM) | 0.58 |
| DMH (11.6 mM) + S9 | 0.58 |
| 4-ABP (300 µM) + S9 | 0.57 |
| Temozolomide (200 µM).1 | 0.57 |
| Formaldehyde (120 µM) | 0.56 |
| MNU (350 µM) | 0.56 |
| 3-NBA (0.025 µM) | 0.55 |
| Temozolomide (200 µM) | 0.53 |
| 1,8-DNP (8 µM) | 0.51 |
| 1,8-DNP (0.125 µM) | 0.47 |
| Mechlorethamine (0.3 µM) | 0.46 |

| | |
|---|---|
| Benzidine (200 µM) | 0.45 |
| DBA (75 µM) + S9 | 0.45 |
| 1,6-DNP (0.09 µM) | 0.45 |
| DBAC (5 µM) + S9 | 0.45 |
| 5-Methylchrysene (1.6 µM) + S9 | 0.44 |
| MX (7 µM) + S9 | 0.43 |
| Furan (100 mM) + S9 | 0.43 |
| BaP (0.39 µM) + S9 | 0.42 |
| DBADE (0.109 µM) | 0.41 |
| PhIP (4 µM) + S9 | 0.40 |
| DBADE (0.0313 µM) | 0.40 |
| BaP (2 µM) + S9 | 0.39 |
| Control | 0.39 |
| BPDE (0.125 µM) | 0.38 |
| *N*-Nitrosopyrrolidine (50 mM) | 0.36 |
| PhIP (3 µM) + S9 | 0.36 |
| Aflatoxin B1 (0.25 µM) + S9 | 0.35 |
| Carboplatin (5 µM) | 0.35 |
| Cisplatin (12.5 µM) | 0.35 |
| Methyleugenol (1.25 mM) | 0.34 |
| Semustine (150 µM) | 0.33 |
| Cisplatin (3.125 µM) | 0.33 |
| Cyclophosphamide (18.75 µM) + S9 | 0.32 |
| Potassium bromate (260 µM) | 0.31 |
| Potassium bromate (875 µM) | 0.30 |
| Ochrotoxin A (0.08 µM) + S9 | 0.26 |
| Simulated solar radiation (1.25 J) | 0.18 |

## References for Supplementary Material

Besaratinia A and Pfeifer GP (2004) Genotoxicity of acrylamide and glycidamide. J Natl Cancer Inst 96(13):1023-1029

Kucab JE, Zou X, Morganella S, Joel M, Nanda AS, Nagy E, Gomez C, Degasperi A, Harris R, Jackson SP, Arlt VM, Phillips DH, Nik-Zainal S (2019) A Compendium of Mutational Signatures of Environmental Agents. Cell 177:821-836 e816

Kucab JE, van Steeg H, Luijten M, Schmeiser HH, White PA, Phillips DH and Arlt VM (2015) TP53 mutations induced by BPDE in Xpa-WT and Xpa-Null human TP53 knock-in (Hupki) mouse
embryo fibroblasts. Mutat Res 773:48-62

Manjanatha MG, Aidoo A, Shelton SD, Bishop ME, McDaniel LP, Lyn-Cook LE and Doerge DR (2006) Genotoxicity of acrylamide and its metabolite glycidamide administered in drinking water to male and female Big Blue mice. Environ Mol Mutagen 47(1):6-17

Manjanatha MG, Guo LW, Shelton SD and Doerge DR (2015) Acrylamide-induced carcinogenicity in mouse lung involves mutagenicity: cII gene mutations in the lung of big blue mice exposed to acrylamide and glycidamide for up to 4 weeks. Environ Mol Mutagen 56(5):446-456

Mei N, McDaniel LP, Dobrovolsky VN, Guo X, Shaddock JG, Mittelstaedt RA, Azuma M, Shelton SD, McGarrity LJ, Doerge DR and Heflich RH (2010) The genotoxicity of acrylamide and glycidamide in big blue rats. Toxicol Sci 115(2):412-421

Nedelko T, Arlt VM, Phillips DH and Hollstein M (2009) TP53 mutation signature supports involvement of aristolochic acid in the aetiology of endemic nephropathy-associated tumours. Int J Cancer 124(4):987-990

Reinbold M, Luo JL, Nedelko T, Jerchow B, Murphy ME, Whibley C, Wei Q and Hollstein M (2008) Common tumour p53 mutations in immortalized cells from Hupki mice heterozygous at codon 72. Oncogene 27(19):2788-2794

vom Brocke J, Krais A, Whibley C, Hollstein MC and Schmeiser HH (2009) The carcinogenic air pollutant 3-nitrobenzanthrone induces GC to TA transversion mutations in human p53 sequences Mutagenesis 24(1):17-23

Whibley C, Odell AF, Nedelko T, Balaburski G, Murphy M, Liu Z, Stevens L, Walker JH, Routledge M and Hollstein M (2010) Wild-type and Hupki (human p53 knock-in) murine embryonic fibroblasts: p53/ARF pathway disruption in spontaneous escape from senescence. J Biol Chem 285(15):11326-11335
